# Aspirin in patients admitted to hospital with COVID-19 (RECOVERY): a randomised, controlled, open-label, platform trial

**DOI:** 10.1016/S0140-6736(21)01825-0

**Published:** 2022-01-08

**Authors:** RECOVERY Collaborative Group, Obbina Abani, Ali Abbas, Fatima Abbas, Mustafa Abbas, Sadia Abbasi, Hakam Abbass, Alfie Abbott, Nabeel Abdallah, Ashraf Abdelaziz, Mohamed Abdelfattah, Bushra Abdelqader, Basir Abdul, Althaf Abdul Rasheed, Ajibode Abdulakeem, Rezan Abdul-Kadir, Abdullah Abdullah, Abdulfatahi Abdulmumeen, Rasheed Abdul-Raheem, Niyaz Abdulshukkoor, Kula Abdusamad, Yazeed Abed El Khaleq, Mai Abedalla, Abeer Ul Amna Abeer Ul Amna, Katrina Abernethy, Adebanke Aboaba, Hani Abo-Leyah, Ahmed Abou-Haggar, Mahmoud Abouibrahim, Miriam Abraham, Tizzy Abraham, Abraheem Abraheem, Judith Abrams, Hyacinth-John Abu, Ahmed Abu-Arafeh, Syed Mohamed Abubacker, Akata Abung, Yaa Aceampong, Amaka Achara, Devikumar Acharya, Sarah Acheampong, Janet Acheson, Andres Acosta, Catherine Acton, Jacqueline Adabie-Ankrah, Fiona Adam, Matthew Adam, Huzaifa Adamali, Carol Adams, Charlotte Adams, Kate Adams, Richard Adams, Tim Adams, Kirsty Adcock, Ade Adebiyi, Ken Adegoke, Vicki Adell, Sherna Adenwalla, Oluwasegun A Adesemoye, Emmanuel O Adewunmi, Joyce Adeyemi, Binay Adhikari, Rina Adhikary, Gabrielle Adkins, Adnan Adnan, John Aeron-Thomas, Debbie Affleck, Carmel Afnan, Muhammad Afridi, Zainab A Aftab, Meenakshi Agarwal, Rachel Agbeko, Chris Agbo, Sunil Aggarwal, Arameh Aghababaie, Shafana Ahamed Sadiq, Mohamed H Ahammed Nazeer, Mohammad Ahmad, Syed Ahmad, Asim Ahmed, Bilal Ahmed, Forizuddin Ahmed, Hamze Ahmed, Iram Ahmed, Irshad Ahmed, Khaled Ahmed, Liban Ahmed, Mahin Ahmed, Maria C Ahmed, Muhammad S Ahmed, Naseer Ahmed, Nausheen Ahmed, Osama Ahmed, Rajia A Ahmed, Rizwan Ahmed, Saif Ahmed, Sammiya Ahmed, Sara Ahmed, Syed Ahmed, Syed Haris Ahmed, Roa Ahmed Ali, Sana Ahmed, Sana Ahmer, Dhiraj Ail, Mark Ainsworth, Myriam Aissa, Lindianne Aitken, Bini Ajay, Abdulakeem Ajibode, Ayesha Ajmi, Nasim Akhtar, Nauman Akhtar, Suha Akili, Oludoyinsola Akindolie, Yinka Akinfenwa, Olugbenga Akinkugbe, Ibrahim Akinpelu, Olugbenro Aktinade, Ahmad Al Aaraj, Asma Al Balushi, Majd Al Dakhola, Aladdin Al Swaifi, Eslam Al-Abadi, Narendra Aladangady, Ayaz Alam, Sajid Alam, Abbas Al-Asadi, Kyriaki Alatzoglou, Paul Albert, Lorraine Albon, Gemma Alcorn, Stephen Alcorn, Aggie Aldana, David Alderdice, Rayan Aldouri, Jonathan Aldridge, Nicolas Aldridge, Ana Alegria, Alison Alexander, John Alexander, Peter D G Alexander, Julyan Al-Fori, Laith Alghazawi, Bahij Al-Hakim, Shams Al-Hity, Ali Ali, Asad Ali, Fawzia R Ali, Jawad Ali, Mariam Ali, Mohammad Ali, Nayab Ali, Oudai Ali, Sakina Ali, Syed Ali, Abid Alina, Fine Aliyuda, Katrin Alizadeh, Maithem Al-Jibury, Saba Al-Juboori, Majid Al-Khalil, Moutaz Alkhusheh, Fiona Allan, Alison Allanson, Robert Allcock, Eireann Allen, Kerry Allen, Louise Allen, Poppy Allen, Rebecca Allen, Sam Allen, Sharon Allen, Simon Allen, Kathryn Allison, Bethan Allman, Lynne Allsop, Hassan Al-Moasseb, Magda Al-Obaidi, Lina Alomari, Akram Al-Rabahi, Bahar Al-Ramadhani, Zayneb Al-Saadi, Inji Alshaer, Rustam Al-Shahi Salman, Warkaq Al-Shamkhani, Bashar Al-Sheklly, Sara Altaf, Mary Alvarez, Maysaa Alzetani, Susan Amamou, Noor Amar, Sakkarai Ambalavanan, Sarah-Jayne Ambler, Robert Ambrogetti, Chris Ambrose, Amir Ameen, Maria R Amezaga, Allison Amin, Amina Amin, Kanish Amin, Syed Amin, Tara Amin, Amjad Amjad, Neelma Amjad, Victoria Amosun, Khaled Amsha, Pugh Amy, Atul Anand, Samantha Anandappa, Julie Anderson, Laura Anderson, Michelle Anderson, Nicola Anderson, Rachel Anderson, Rory Anderson, Wendy Anderson, Prematie Andreou, Angela Andrews, Antonette Andrews, Jill Andrews, Kanayochukwu Aneke, Andrew Ang, Wan Wei Ang, Tammy Angel, Aramburo Angela, Paola Angelini, Lazarus Anguvaa, Oleg Anichtchik, Millicent Anim-Somuah, Krishnan Aniruddhan, Jessica Annett, Patrick James Anstey, Rebekah Anstey, Alpha Anthony, Aaron Anthony-Pillai, Philip Antill, Zhelyazkova Antonina, Varghese Anu, Muhammad Anwar, Aristeidis Apostolopoulos, Sarah Appleby, Diane Appleyard, Maia Far Aquino, Bianca Araba, Samuel Aransiola, Mariana Araujo, Ann Archer, Denise Archer, Simon Archer, Christian Ardley, Ana-Maria Arias, Ryoki Arimoto, Charlotte Arkley, Charlotte Armah, Ilianna Armata, Adam Armitage, Ceri Armstrong, Maureen Armstrong, Sonia Armstrong, Philippa Armtrong, Heike Arndt, Clare Arnison-Newgass, David Arnold, Rachael Arnold, Dhawal Arora, Kavan Arora, Pardeep Arora, Rishi Arora, Arslam Arter, Ayush Arya, Rita Arya, Denisa Asandei, Adeeba Asghar, Catherine Ashbrook-Raby, Helen Ashby, Jan Ashcroft, John Ashcroft, Samuel Ashcroft, Georgia Asher, Ayesha Ashfaq, Abdul Ashish, Sally Ashman-Flavell, Sundar Ashok, Abd-El-Aziz Ashour, Muhammad Zubair Ashraf, Saima Ashraf, Mohammad Bilal Ashraq, Deborah Ashton, Susan Ashton, Andrew Ashworth, Rebecca Ashworth, Arshia Aslam, Harshini Asogan, Atif Asrar, Omar Assaf, Raine Astin-Chamberlain, Deborah Athorne, Billie Atkins, Christopher Atkins, Stacey Atkins, John Atkinson, Vicki Atkinson, Brygitta Atraskiewicz, Abdul Ahmad Attia, Paula Aubrey, Avinash Aujayeb, Aye Chan Thar Aung, Hnin Aung, Kyaw Thu Aung, Yin Aung, Zaw Myo Aung, Emily Austin, Karen Austin, Abdusshakur Auwal, Miriam Avery, Nicholas Aveyard, Joanne Avis, Georgina Aviss, Cristina Avram, Paula Avram, Gabriel Awadzi, Atia Awan, Aszad Aya, Eman Ayaz, Amanda Ayers, Jawwad Azam, Mohammed Azharuddin, Ghazala Aziz, N Aziz, Ali Azkoul, Ashaari Azman Shah, Giada Azzopardi, Hocine Azzoug, Fiyinfoluwa Babatunde, Melvin Babi, Babiker Babiker, Gayna Babington, Matthew Babirecki, Marta Babores, Adetona O Babs-Osibodu, Sammy Bacciarelli, Roudi Bachar, Gina Bacon, Jenny Bacon, Bibi Badal, Gurpreet Rani Badhan, Shreya Badhrinarayanan, Joseph P Bae, Alice Baggaley, Amy Baggott, Graham Bagley, Dinesh Bagmane, Lynsey Bagshaw, Kasra Bahadori, James Bailey, Katie Bailey, Lindsey Bailey, Liz Bailey, Morgan Bailey, Pippa Bailey, Sarah Bailey, Hamish Baillie, J Kenneth Baillie, Jennifer Bain, Vikram Bains, David Baird, Kevin Baird, Susan Baird, Tracy Baird, Yolanda Baird, Aiysha Bajandouh, Evelyn Baker, Johanne Baker, Josephine Baker, Kenneth Baker, Rebecca Baker, Terri-Anne Baker, Victoria Baker, Hugh Bakere, Nawar Bakerly, Michelle Baker-Moffatt, Nauman Bakhtiar, Panos Bakoulas, Niranjan Balachandran, Andrea Balan, Theodosios Balaskas, Madhu Balasubramaniam, Alison Balcombe, Alexander Baldwin, Ashley Baldwin, Caron Baldwin, Danielle Baldwin, Rebekah Baldwin-Jones, James Balfour, Matthew Ball, K Ballard, Ismael Balluz, Craig Balmforth, Emese Balogh, Amir Baluwala, Gabby Bambridge, Alasdair Bamford, Amy Bamford, Peter Bamford, Adefunke Bamgboye, Elizabeth Bancroft, Hollie Bancroft, Joyce Banda, Krishna Bandaru, Srini Bandi, Nageswar Bandla, Somaditya Bandyopadhyam, Amit Banerjee, Ritwik Banerjee, Harrison Banks, Luke Banks, Paul Banks, Oliver Bannister, Laura Banton, Tran Bao, Mariamma Baptist, Tanya Baqai, Ananya Mouli Baral, Desislava Baramova, Russel Barber, Emma Barbon, Monica Barbosa, Jamie Barbour, Alexander Barclay, Claire Barclay, George Bardsley, Stephanie Bareford, Shahedal Bari, Morris Barimbing, Amy Barker, Debbie Barker, Helen Barker, Joseph Barker Barker, Leon Barker, Oliver Barker, Kerry Barker-Williams, Sinha Barkha, Juliana Barla, Gavin Barlow, Richard Barlow, Valerie Barlow, James Barnacle, James Barnacle, Alex Barnard, Debi Barnes, Nicky Barnes, Theresa Barnes, Calum Barnetson, Amy Barnett, Ashton Barnett-Vanes, William Barnsley, Andrew Barr, David Barr, Shaney Barratt, Manuella Barrera, Amy Barrett, Fiona Barrett, Jessica Barrett, Jazz Bartholomew, Claire Bartlett, Georgina Bartlett, Greg Barton, Jill Barton, Lorna Barton, Rachael Barton, Rosaleen Baruah, Sonia Baryschpolec, Archana Bashyal, Betsy Basker, Buddha Basnyat, Ayten Basoglu, John Bassett, G Bassett, Chris Bassford, Bengisu Bassoy, Victoria Bastion, Anup Bastola, Anupam Basumatary, Tristan Bate, Harry J Bateman, Kathryn Bateman, Vhairi Bateman, Eleanor Bates, Hayley Bates, Michelle Bates, Simon Bates, Sally Batham, Ana Batista, Amit Batla, Dushyant Batra, Harry Batty, Thomas Batty, Miranda Baum, Rachel Baumber, Carina Bautista, Fareha Bawa, Fatima S Bawani, Simon Bax, Matt Baxter, Nicola Baxter, Zachary Baxter, Hannah Bayes, Farid Bazari, Rohit Bazaz, Ahmad Bazli, Laura Beacham, Wendy Beadles, Philip Beak, Andy Beale, Jack Bearpark, Karen Beaumont, Dawn Beaumont-Jewell, Theresa Beaver, Sarah Beavis, Christy Beazley, Sarah Beck, Virginia Beckett, Rosie Beckitt, Heidi Beddall, Seonaid Beddows, Deborah Beeby, Gail Beech, Michelle Beecroft, Sally Beer, Jane Beety, Gabriela Bega, Alison Begg, Susan Begg, Sara Beghini, Ayesha Begum, Salman Begum, Selina Begum, Teresa Behan, Roya Behrouzi, Jon Beishon, Claire Beith, James Belcher, Holly Belfield, Katherine Belfield, Ajay Belgaumkar, Dina Bell, Gareth Bell, Gillian Bell, Lauren Bell, Louise Bell, Nicholas Bell, Pippa Bell, Stephanie Bell, Jennifer L Bell, Jennifer Bellamu, Mary Bellamy, Arianna Bellini, Amanda Bellis, Fionn Bellis, Lesley Bendall, Naveena Benesh, Nicola Benetti, Leonie Benham, Guy Benison-Horner, Ann Bennett, Caroline Bennett, Gillian Bennett, Kristopher Bennett, Lorraine Bennett, Sara Bennett, Karen Bennion, Vivienne Benson, Andrew Bentley, James Bentley, Ian Benton, Eva Beranova, Matthew Beresford, Colin Bergin, Malin Bergstrom, Jolanta Bernatoniene, Thomas Berriman, Zoe Berry, Kimberley Best, Ans-Mari Bester, Yvonne Beuvink, Emily Bevan, Sarah Bevins, Tom Bewick, Andrew Bexley, Sonay Beyatli, Fenella Beynon, Arjun Bhadi, Sanjay Bhagani, Shiv Bhakta, Rekha Bhalla, Khushpreet Bhandal, Kulbinder Bhandal, Ashwin Bhandari, Sangam Bhandari, Aashutosh Bhanot, Ravina Bhanot, Prashanth Bhat, Nikhil Bhatia, Rahul Bhatnagar, Karan Bhatt, Janki Bhayani, Deepika Bhojwani, Salimuzzaman Bhuiyan, Anna Bibby, Fatima Bibi, Naheeda Bibi, Salma Bibi, Tihana Bicanic, Sarah Bidgood, Julie Bigg, Sarah Biggs, Alphonsa Biju, Andras Bikov, Sophie Billingham, Jessica Billings, Alice Binns, Muhammad BinRofaie, Oliver Bintcliffe, Catherine Birch, Jenny Birch, Katherine Birchall, Sam Bird, Sumedha Bird, Mark Birt, Kilanalei Bishop, Linda Bishop, Lisa Bishop, Karen Bisnauthsing, Nibedan Biswas, Sahar Biuk, Karen Blachford, Ethel Black, Helen Black, Karen Black, Mairead Black, Polly Black, Hayley Blackgrove, Bethan Blackledge, Joanne Blackler, Samantha Blackley, Helen Blackman, Caroline Blackstock, Francesca Blakemore, Helen Blamey, Alison Bland, Sujata Blane, Simon Blankley, Parry Blaxill, Katie Blaylock, Jane Blazeby, Natalie Blencowe, Ben Bloom, Jack Bloomfield, Angela Bloss, Hannah Bloxham, Louise Blundell, Andrew Blunsum, Mark Blunt, Ian Blyth, Kevin Blyth, Andrew Blythe, Karen Blythe, Marilyn Boampoaa, Boniface Bobie, Karen Bobruk, Pritesh Bodalia, Neena Bodasing, Tanya Bodenham, Gabriele Boehmer, Marta Boffito, Kristyna Bohmova, Sumit Bokhandi, Maria Bokhar, Saba Bokhari, Sakina Bokhari, Syed O Bokhari, Ambrose Boles, Charlotte Bond, Helena Bond, Stuart Bond, Thomas Bond, Alice Bone, Georgia Boniface, Lizzy Bonney, Joanne Borbone, Naomi Borman, Fiona Bottrill, Laura Bough, Hayley Boughton, Zoe Boult, Miriam Bourke, Stephen Bourke, Michelle Bourne, Rachel Bousfield, Lucy Boustred, Alexandra Bowes, Amy Bowes, Philip Bowker, Louise Bowman, Simon Bowman, Rachel Bowmer, Angie Bowring, Helen Bowyer, Jenny Boyd, Laura Boyd, Namoi Boyle, Pauline Boyle, Rosalind Boyle, Louise Boyles, Leanna Brace, Jodie Bradder, Clare Jane Bradley, Pamela Bradley, Patrick Bradley, Paul Bradley, Joanne Bradley-Potts, Lynne Bradshaw, Zena Bradshaw, Rebecca Brady, Shirin Brady, Denise Braganza, Marie Branch, Thomas Brankin-Frisby, Jamie Brannigan, Louise Brassington, Sophie Brattan, Fiona Bray, Nancy Bray, Manny Brazil, Lucy Brear, Tracy Brear, Stephen Brearey, Laura Bremner, Morwenna Brend, Giovanna Bretland, Chris Brewer, Gavin Bridgwood, Sara Brigham, John Bright, Chris Brightling, Lutece Brimfield, Elaine Brinkworth, Robin Brittain-Long, Vianne Britten, Lauren Broad, Sarah Broad, Rosie Broadhurst, Andrew Broadley, Marie Broadway, Christopher Brockelsby, Megan Brocken, Tomos Brockley, Mary Brodsky, Fiona Brogan, Liz Brohan, Felicity Brokke, Jacob Brolly, David Bromley, Hannah Brooke-Ball, Verity Brooker, Matthew Brookes, Deirdre Brooking, Alison Brooks, Karen Brooks, Nicole Brooks, Philip Brooks, Rachel Brooks, Sophie Brooks, Natalie Broomhead, Chloe Broughton, Nathaniel Broughton, Matt Brouns, Alison Brown, Ammani Brown, Carly Brown, Catrin Brown, Ellen Brown, Heather Brown, Janet Brown, Louise Brown, Niall Brown, Pauline Brown, Richard Brown, Robert Brown, Steven Brown, Thomas Brown, Bria Browne, Charlotte Browne, Duncan Browne, Mitchell Browne, Stephen Brownlee, Alba Brraka, David Bruce, Johanna Bruce, Michelle Bruce, Wojciech Brudlo, Nigel Brunskill, Alan Brunton, Margaret Brunton, Meera Bryant, April Buazon, Maya H Buch, Ruaridh Buchan, Ruaridh Buchanan, Danielle Buche, Amanda Buck, Matthew Buckland, Laura Buckley, Philip Buckley, Sarah Buckley, Carol Buckman, George Bugg, Ramadan Bujazia, Marwan Bukhari, Shanze Bukhari, Richard Bulbulia, Alex Bull, Damian Bull, Rhian Bull, Thomas Bull, Naomi Bulteel, Kasun Bumunarachchi, Roneleeh Bungue-Tuble, Caroline Burchett, Dorota Burda, Christy Burden, Thomas G Burden, Mika Burgess, Richard Burgess, Sophia Burgess, Adrian Burman, Sara Burnard, Caroline Burnett, Amy Burns, Collette Burns, James Burns, Karen Burns, Daniel Burrage, Kate Burrows, Claire Burston, Ben Burton, Fiona Burton, Matthew Burton, Deborah Butcher, Aaron Butler, Jessica Butler, Joanne Butler, Joshua Butler, Peter Butler, Susan Butler, Al-Tahoor Butt, Mohammad M Butt, Caryl Butterworth, Nicola Butterworth-Cowin, Robert Buttery, Tom Buttle, Heather Button, Daniel Buttress, Jane Byrne, Wendy Byrne, Victoria Byrne-Watts, Amanda Cabandugama, Ruth Cade, Anthony Cadwgan, Ajeng Cahyareny, Donna Cairney, James Calderwood, Darren Caldow, Giorgio Calisti, Debbie Callaghan, Jennifer Callaghan, Claire Callens, Donaldson Callum, Caroline Calver, Melissa Cambell-Kelly, Tracey Camburn, David Richard Cameron, Eleanor Cameron, Fraser Cameron, Sheena Cameron, Christian Camm, Renee F D Cammack, Alison Campbell, Amy Campbell, Barbara Campbell, Bridget Campbell, Debbie Campbell, Helen Campbell, Hilary Campbell, Jonathan Campbell, Mark Campbell, Robyn Campbell, Wynny Campbell, Quentin Campbell Hewson, Julie Camsooksai, Lisa Canclini, Shaula Mae Candido, Janie Candlish, Cielito Caneja, Johnathon Cann, Ruby Cannan, Emma Cannon, Michael Cannon, Petra Cannon, Vivienne Cannons, Jane Cantliff, Ben Caplin, Santino Capocci, Noemi Caponi, Angelika Capp, Thomas Capstick, Mary Cardwell, Rachel Carey, Simon Carley, Tammy Carlin, Samantha Carmichael, Mandy Carnahan, Charlotte Caroline, Jodi Carpenter, Sharon Carr, Anna Carrasco, Zoe Carrington, Paul Carroll, Jonathan Carter, Michael Carter, Paul Carter, Penny Carter, Douglas Cartwright, Jo-Anne Cartwright, Claire Carty, Jaime Carungcong, Susan Casey, Annie Cassells, Barbara Cassimon, Teresa Castiello, Gail Castle, Bridget Castles, Melanie Caswell, Ana Maria Catana, Heidi Cate, Susanne Cathcart, Katrina Cathie, Christine Catley, Laura Catlow, Matthew Caudwell, Anna Cavazza, Luke Cave, Simon Cavinato, Frianne Cawa, Kathryn Cawley, Chloe Caws, Hankins Cendl, Hannah Century, Jeva Cernova, Mansur Cesay, Ed Cetti, Stephanie Chabane, Manish Chablani, Cathleen Chabo, David Chadwick, Julie Chadwick, Robert Chadwick, Ela Chakkarapani, Arup Chakraborty, Mallinath Chakraborty, Mollika Chakravorty, Bimal Chalise, James Chalmers, Richard Chalmers, Georgina Chamberlain, Sarah Chamberlain, Emma Chambers, Jonathan Chambers, Lucy Chambers, Naomi Chambers, Alex Chan, Carmen Chan, Cheuk Chan, Evelyn Chan, Kayen Chan, Kimberley Chan, Ping Chan, Rebekah (Pui-Ching) Chan, Xin Hui Chan, Chris Chandler, Heidi Chandler, Kim Jessica Chandler, Stuart Chandler, Zoe Chandler, Sumit Chandra, Navin Chandran, Badrinathan Chandrasekaran, Yvonne Chang, Josephine Chaplin, Graeme Chapman, John Chapman, Katie Chapman, Laura Chapman, Lianne Chapman, Polly Chapman, Timothy Chapman, Lucy C Chappell, Amanda Charalambou, Bethan Charles, Dianne Charlton, Kevin Chatar, Calvin Chatha, Ritesh Chaube, Muhammad Yafaa Naveed Chaudhary, Iram Chaudhry, Nazia Chaudhuri, Muhammad Chaudhury, Anoop Chauhan, Ruchi Singh Chauhan, Nicola Chavasse, Vipal Chawla, Lindsay Cheater, James Cheaveau, Charlotte Cheeld, Michelle Cheeseman, Fang Chen, Hui Min Chen, Terence Chen, Lok Yin Cheng, Zhihang Cheng, Helen Chenoweth, Chun How Cheong, Shiney Cherian, Mary Cherrie, Helen Cheshire, Chee Kay Cheung, Elaine Cheung, Michelle Cheung, Victor Chew, Claire Cheyne, Swati Chhabra, Wei Ling Chia, Eric Chiang, Angela Chiapparino, Rosavic Chicano, Zviedzo Agenia Chikwanha, Sam Chilcott, Phillipa Chimbo, KokWai Chin, Wen Jie Chin, Rumbidzai Chineka, Amol Chingale, Heather Chisem, Claire Chisenga, Ben Chisnall, Carolyn Chiswick, Sunder Chita, Nihil Chitalia, Matthew Chiu, Brenda Chivima, Catherine Chmiel, Soha Choi, Willy Choon Kon Yune, Vandana Choudhary, Sarah Choudhury, Bing-Lun Chow, Mahibbur Chowdhury, Shahid Chowdhury, Victoria Christenssen, Peter Christian, Alexander Christides, Fiona Christie, Daniel Christmas, Thereza Christopherson, Mark Christy, Paris Chrysostomou, Yunli Chua, Dip Chudgar, Richard Chudleigh, Srikanth Chukkambotla, Michael Eze Chukwu, Izu Chukwulobelu, Chi Y Chung, Elaine Church, Sara R Church, David Churchill, Nicole Cianci, Paola Cicconi, Paola Cinardo, Zdenka Cipinova, Bessie Cipriano, Sarah Clamp, Melanie Clapham, Edel Clare, Sarbjit Clare, Andrew Clark, Charlotte Clark, Diane Clark, Felicity Clark, Gabrielle Clark, James Clark, Katherine Clark, Kaylea Clark, Louise Clark, Lucy Clark, Matthew Clark, Patricia Clark, Richard Clark, Thomas Clark, Zoe Clark, Andrea Clarke, Paul Clarke, Robert Clarke, Roseanne Clarke, Samantha Clarke, Sheron Clarke, Alleyna Claxton, Kate Clay, Elizabeth Clayton, Olivia Clayton, Jill Clayton-Smith, Chris Cleaver, Carlota Clemente de la Torre, Jayne Clements, Suzanne Clements, Rachael Clifford, Sarah Clifford, Amelia Clive, Jonathan Clouston, Samantha Clueit, Andrea Clyne, Michelle Coakley, Peter Gerald Leyland Coakley, Kathryn Cobain, Alexandra Cochrane, Patricia Cochrane, Maeve Cockerell, Helen Cockerill, Shirley Cocks, Rachel Codling, Adam Coe, Samantha Coetzee, David Coey, Danielle Cohen, Jonathan Cohen, Oliver Cohen, Mike Cohn, Louise Coke, Olutoyin Coker, Nicholas Colbeck, Roghan Colbert, Esther Cole, Jade Cole, Joby Cole, Garry Coleman, Matt Coleman, Holly Coles, Macleod Colin, Alicia Colino-Acevedo, Julie Colley, Dawn Collier, Heather Collier, Paul Collini, Emma Collins, Jaimie Collins, Joanne Collins, Nicola Collins, Sally Collins, Vicky Collins, Andrew Collinson, Bernadette Collinson, Jennifer Collinson, Matthew Collis, Madeleine Colmar, Hayley Emma Colton, James Colton, Katie Colville, Carolyn Colvin, Edward Combes, David Comer, Alison Comerford, Dónal Concannon, Robin Condliffe, Lynne Connell, Natalie Connell, Karen Connelly, Gavin Connolly, Emma Connor, Antonia Conroy, Veronica Conteh, Rory Convery, Francesca Conway, Grainne Conway, Rhiannon Conway, Jo-Anna Conyngham, Colette Cook, Eloise Cook, Gemma Cook, Helen Cook, Julie Cook, Danielle Cooke, Graham Cooke, Katrina Cooke, Tim Cooke, Adele Cooper, Chris Cooper, David Cooper, Helen Cooper, Jamie Cooper, Lauren Cooper, Nick Cooper, Rowena Cooper, Thomas Cope, Sinead Corbet, Carolyn Corbett, John Corcoran, Chris Cordell, Jessica Cordle, Alasdair Corfield, John Corless, Alison Corlett, Joe Cornwell, Michael Cornwell, Diana Corogeanu, Mirella Corredera, Ruth Corrigan, Rita Corser, Denise Cosgrove, Tracey Cosier, Patricia Costa, Charlie Coston, Susannah Cotgrove, Zoe Coton, Lisa-Jayne Cottam, Rhiannon Cotter, Donna Cotterill, Caroline Cotton, Andrew Coull, James Coulson, David Counsell, David Counter, Cherry Coupland, Ellie Courtney, Julia Courtney, Rebecca Cousins, Alexander Cowan, Elena Cowan, Richard Cowell, Louise Cowen, Steve Cowman, Amanda Cowton, Ellie Cox, Giles Cox, Karina Cox, Miriam Cox, Karen Coy, Andrea Cradduck-Bamford, Victoria Craig, Felicity Craighead, Matthew Cramp, Jacolene Crause, Angie Crawford, Emma Crawford, Isobel Crawford, Sarah Crawshaw, Ben Creagh-Brown, Andrew Creamer, Joanne Cremona, Saveria Cremona, Janet Cresswell, Mark Cribb, Charles Crichton, Declan Crilly, Lauren Crisp, Nikki Crisp, Dominic Crocombe, Maria Croft, Jennifer Crooks, Harriet Crosby, Elizabeth Cross, Tim Cross, Amy Crothers, Stephen Crotty, Susan Crouch, Madeleine Crow, Amanda Crowder, Kate Crowley, Teresa Crowley, Rebecca Croysdill, Callum Cruickshank, Conor Cruickshank, Irena Cruickshank, James Cruise, Carina Cruz, Trino Cruz Cervera, Dominic Cryans, Guanguo Cui, Helen Cui, Lorraine Cullen, Gillian Cummings-Fosong, Victoria Cunliffe, Neil Cunningham, Nicola Cunningham, Jason Cupitt, Hollie Curgenven, Gerens Curnow, David Curran, Simon Curran, Craig Currie, Jacqueline Currie, Scarlett Currie, Jonathan Curtis, Katrina Curtis, Olivia Curtis, Thomas Curtis, Rebecca Cuthbertson, Sean Cutler, Marta Czekaj, Patrycja Czylok, Joana da Rocha, Andrew Dagens, Helen Daggett, Jacqui Daglish, Sandeep Dahiya, Anne Dale, Katie Dale, Michaela Dale, Sam Dale, Jolyon Dales, Helen Dalgleish, Helen Dallow, Dermot Dalton, Zoe Daly, Akila Danga, Amelia Daniel, Priya Daniel, Allison Daniels, Adela Dann, Sandra Danso-Bamfo, Alex Darbyshire, Janet Darbyshire, Paul Dargan, Paul Dark, Kate Darlington, Tom Darton, Guledew Darylile, Manjusha Das, Sukamal Das, Martin Daschel, Joanne Dasgin, Dibyendu Datta, Anna Daunt, Emily Davenport, Mark Davey, Miriam Davey, Molly Davey, Mini David, Alexander Davidson, Laura Davidson, Neil Davidson Davidson, Richard Davidson, Albert Davies, Amanda Davies, Amy Davies, Angela Davies, Carolyn Davies, Catrin Davies, Drew Davies, Elaine Davies, Ffyon Davies, Helen Davies, Jim Davies, Karen Davies, Kelly Davies, Kim Davies, Louisa Davies, Matthew Davies, Michelle Davies, Nina Davies, Owen Davies, Patrick Davies, Rachel Davies, Rhys Davies, Ruth Davies, Sarah Davies, Simon Davies, Gwyneth Davis, Illinos Davis, Julie-Ann Davis, Katherine Davis, Peter Davis, Alexander Davison, Christine Dawe, H Dawe, Mark Dawkins, Danielle Dawson, Elizabeth Dawson, Joy Dawson, Susan Dawson, Tom Dawson, Andrew Daxter, Andrew Day, Jacob Day, Jeremy N Day, Jamie D'Costa, Parijat De, Duneesha de Fonseka, Toni de Freitas, Frederico De Santana Miranda, Eleanor de Sausmarez, Shanika de Silva, Thushan de Silva, Jessica De Sousa, Paulo De Sousa, James de Souza, Anthony de Soyza, Natasha de Vere, Johannes de Vos, Bethan Deacon, Sharon Dealing, Anna Dean, Julie Dean, Katrina Dean, Stephen Dean, Tessa Dean, Jill Deane, James Dear, Effie Dearden, Catherine Deas, Samuel Debbie, Gabor Debreceni, Vashist Deelchand, Matthew Deeley, Joanne Deery, Emmanuel Defever, Manuela Del Forno, Arnold Dela Rosa, Amanda Dell, Carrie Demetriou, David DeMets, Jane Democratis, Jacqueline Denham, Emmanuelle Denis, Laura Denley, Craig Denmade, Kathy Dent, Martin Dent, Elise Denton, Tom Denwood, Nishigandh Deole, Darshita Depala, Maria Depante, Susan Dermody, Amisha Desai, Asmita Desai, Purav Desai, Sanjeev Deshpande, Vai Deshpande, Sirjana Devkota, Usha Devkota, Prakash Dey, Vishal Dey, Rogin Deylami, Kevin Dhaliwal, Sundip Dhani, Amandeep Dhanoa, Mili Dhar, Devesh Dhasmana, Ekanjali Dhillon, Reiss Dhillon, Meghnath Dhimal, Priya Dias, Stephanie Diaz, Kayleigh Diaz-Pratt, Debbie Dickerson, Pamela Dicks, Stuart Dickson, Sean Dillane, Sarah Diment, Paul Dimitri, Thai Ha Dinh, Alex Dipper, Laura Dirmantaite, Lisa Ditchfield, Sarah Diver, Lavanya Diwakar, Caroline Dixon, Giles Dixon, Brice Djeugam, Petr Dlouhy, Paul Dmitri, Laurence Dobbie, Marinela Dobranszky Oroian, Charlotte Dobson, Lee Dobson, Marie Docherty, David Dockrell, James Dodd, Jackie Dodds, Rebecca Dodds, Steve Dodds, Richi Dogra, Erin Doherty, Warren Doherty, Yumiko Doi, Iain Doig, Eleanor Doke, Daniel Dolan, Mark Dolman, Rozzie Dolman, Lisa Donald, Callum Donaldson, Christopher Donaldson, Denise Donaldson, Gillian Donaldson, Kate Donaldson, Joanne Donnachie, Christopher Donnelly, Eilish Donnelly, Ronan Donnelly, Aravindhan Donohoe, Gemma Donohoe, Bryan Donohue, Sinead Donton, Emma Dooks, Grainne Doran, Kane Dorey, Sharon Dorgan, Moonira Dosani, Davinder Dosanjh, Paula Dospinescu, Katie Douglas, Jonathan Douse, Lucy Dowden, Michelle Dower, Sud Dowling, Nicola Downer, Charlotte Downes, Rob Downes, Thomas Downes, Damian Downey, Robert Downey, Louise Downs, Simon Dowson, Cornel Dragan, Cristina Dragos, Maire Drain, Chelsea Drake, Victoria Drew, Olivia Drewett, Celine Driscoll, Helena Drogan, Graham Drummond, Ronald Druyeh, Jack Dryburgh-Jones, Simon Drysdale, An Du Thinh, Hazel Dube, Judith Dube, Stephen Duberley, Hayley Duckles-Leech, Nicola Duff, Emma Duffield, Helen Duffy, Lionel Dufour, Annette Duggan, Parveen Dugh, Janice Duignan, Simon Dummer, Andrew Duncan, Christopher Duncan, Fullerton Duncan, Gregory Duncan, Stephanie Dundas, Alessia Dunn, Charlotte Dunn, Damian Dunn, Laura Dunn, Paul Dunn, Charlene Dunne, Karen Dunne, Fiona Dunning, Aidan Dunphy, Venkat Duraiswamy, Beatriz Duran, Ingrid DuRand, Natalie Duric, Alison Durie, Emily Durie, Hannah Durrington, Haris Duvnjak, Akshay Dwarakanath, Laasya Dwarakanath, Ellen Dwyer, Claudia Dyball, Kristyn Dyer, Harvey Dymond, Tom Dymond, Chris Eades, Laura Eagles, Joanne Early, Melissa Earwaker, Nicholas Easom, Clare East, Amy Easthope, Fraser Easton, Ruth Eatough, Beate Ebert, Oluwadamilola Ebigbola, Martin Ebon, Sinan Eccles, Chloe Eddings, Michael Eddleston, Maureen Edgar, Katharine Edgerley, Nicholas Edmond, Mary Edmondson, Tracy Edmunds, Alexandra Edwards, Catherine Edwards, Joy Edwards, Kennedy Edwards, Mandy Edwards, Jenny Eedle, Dawn Egginton, Loveth Ehiorobo, Sarah Eisen, Ugochukwu Ekeowa, Mohamed Ekoi, Ayomide Ekunola, Soha El Behery, Mohamed Elbeshy, Kate El-Bouzidi, Jennifer Elder, Mohammed El-Din, Diana Eleanor, Ibrahim Eletu, Eman Elfar, Mayy Magdy Elgamal, Amr Elgohary, Stellios Elia, Jennifer Elias, Tania Elias, Nadia Elkaram, Andrew Victor Elkins, Julie Ellam, Nikki Ellard, Laura Nicola Ellerton, Lucy Elliot, Amy Elliott, Fiona Elliott, Kerry Elliott, Scott Elliott, Annie Ellis, Christine Ellis, Kaytie Ellis, Tak-Yan Ellis, Yvette Ellis, Rahma Elmahdi, Einas Elmahi, Hannah-May Elmasry, Najla Elndari, Omer Elneima, Mohamed Elokl, Ahmed Elradi, Mohamed Elsaadany, Sally El-Sayeh, Hana El-Sbahi, Tarek Elsefi, Karim El-Shakankery, Hosni El-Taweel, Sarah Elyoussfi, Jonathan Emberey, Jonathan R Emberson, John Emberton, Julian Emmanuel, Ingrid Emmerson, Michael Emms, Florence Emond, Marieke Emonts, Nicu Enachi, Angila Engden, Katy English, Emma Entwistle, Hene Enyi, Marios Erotocritou, Peter Eskander, Hanif Esmail, Brynach Evans, Chris Evans, Debra Evans, Gail Evans, Gareth Evans, Jennifer Evans, Jolanta Evans, Lisa Evans, Lynn Evans, Mim Evans, Morgan Evans, Ranoromanana Evans, Teriann Evans, Terry J Evans, Caroline Everden, Serenydd Everden, Hayley Evison, Lynsey Evison, Jacqueline Faccenda, Leila Fahel, Youstina Fahmay, Sara Fairbairn, Terry Fairbairn, Andy Fairclough, Louise Fairlie, Mark Fairweather, Anne Fajardo, Naomi Falcone, Euan Falconer, John Fallon, Andrea Fallow, David Faluyi, Victoria Fancois, Qayyum Farah, Novin Fard, Leila Fares, Amr Farg, Adam Farmer, Katie Farmer, Toni Farmery, Samantha Farnworth, Faiyaz Farook, Hadia Farooq, Sidrah Farooq, Fiona Farquhar, Aaron Farrell, Barbara Farrell, James Farthing, Syeda Farzana, Rahmatu Fasina, Azam Fatemi, Mina Fatemi, Nibah Fatimah, Maria Faulkner, Saul N Faust, Joe Fawke, Sinmidele Fawohunre, Abul Fazal, Simon Fearby, Alex Feben, Federico Fedel, Daria Fedorova, Christopher Fegan, Mae Felongco, Lynsey Felton, Tim Felton, Kate Fenlon, Andrea Fenn, Isabelle Fenner, Ciara Fenton, Melisa Fenton, Cameron Ferguson, Jenny Ferguson, Kathryn Ferguson, Katie Ferguson, Susan Ferguson, Susie Ferguson, Victoria Ferguson, Denzil Fernandes, Candida Fernandez, Eduardo Fernandez, Maria Fernandez, Sonia Fernandez Lopez, Callum Jeevan Fernando, Ahmed Feroz, Pietro Ferranti, Thais Ferrari, Eleanor Ferrelly, Alexandra Ferrera, Emma Ferriman, Nicholas Fethers, Ben Field, Janet Field, Rebecca Field, Karen Fielder, Lindsey Fieldhouse, Andra Fielding, Julie Fielding, Sarah Fielding, Asma Fikree, Sarah Ann Filson, Sarah Finbow, Debbie Finch, Joanne Finch, Laurie Finch, Natalie Fineman, Lauren Finlayson, Adam Finn, Joanne Finn, Clare Finney, Sofia Fiouni, Jo Fiquet, James Fisher, Neil Fisher, Daniel Fishman, Krystofer Fishwick, Fiona Fitzgerald, Jan Flaherty, Michael Flanagan, Charles Flanders, Julie Fleming, Lucy Fleming, Paul Fleming, William Flesher, Alison Fletcher, Jonathan Fletcher, Lucy Fletcher, Simon Fletcher, Sophie Fletcher, Karen Flewitt, Christopher Flood, Ian Floodgate, Vincent Florence, Sharon Floyd, Rachel Flynn, Claire Foden, Adama Fofana, Georgina Fogarty, Paul Foley, Linda Folkes, Daniela Mock Font, Aiwyne Foo, Jane Foo, Andrew Foot, Jayne Foot, Jane Forbes, Jamie Ford, Jennifer Foreman, Caroline Fornolles, Adam Forrest, Ellie Forsey, Miranda Forsey, Thomas Forshall, Elliot Forster, Julian Forton, Emily Foster, Joseph Foster, Rachel Anne Foster, Tracy Foster, Angela Foulds, Ian Foulds, Folakemi Fowe, Emily Fowler, Robert Fowler, Stephen Fowler, Claire Fox, Heather Fox, Jonathan Fox, Lauren Fox, Natalie Fox, Olivia Fox, Simon Fox, Sarah-Jane Foxton, Rebecca Frake, Alex Francioni, Olesya Francis, Rebecca Francis, Sarah Francis, Theodora Francis-Bacon, Helen Frankland, Jessica Franklin, Catherine Fraser, Sharon Frayling, Martyn Fredlund, Carol Freeman, Elaine Freeman, Hannah Freeman, Nicola Freeman, Clare Freer, Eleanor French, Matthew Frise, Renate Fromson, Claire Froneman, Adam Frosh, John Frost, Victoria Frost, Oliver Froud, Rachel Frowd, Arun Fryatt, Bridget Fuller, Liz Fuller, Tracy Fuller, Duncan Fullerton, Carrie Fung, Gayle Fung, Sarah Funnell, John Furness, Andrew Fyfe, Nytianandan G, Elizabeth Gabbitas, Claire Gabriel, Zoë Gabriel, Hadiza Gachi, Joshua Gahir, Sarveen Gajebasia, Katarzyna Gajewska-Knapik, Christopher Gale, Hugo Gale, Rebecca Gale, Swetha Gali, Bernadette Gallagher, Jude Gallagher, Rosie Gallagher, William Gallagher, Joanne Galliford, Catherine Galloway, Chris Galloway, Emma Galloway, Jacqui Galloway, James Galloway, Laura Gamble, Liz Gamble, Brian Gammon, Jaikumar Ganapathi, Ramesh Ganapathy, Kaminiben Gandhi, Sarah Gandhi, Usha Ganesh, Abrar Gani, Emma-James Garden, Antoni Dariusz Gardener, Emma Gardiner, Michael Gardiner, Phil Gardiner, Siobhan Gardiner, Caroline Gardiner-Hill, Jonathan Gardner, Mark Garfield, Atul Garg, Nathan Garlick, Justin Garner, Lucie Garner, Zoe Garner, Rosaline Garr, Florence Garty, Rachel Gascoyne, Hyeriju Gashau, Aoife Gatenby, Erin Gaughan, Alok Gaurav, Mariana Gavrila, Jane Gaylard, Emma Gaywood, Catherine Geddie, Ian Gedge, Sarah Gee, Minerva Gellamucho, Karzan Gelly, L Gelmon, Leila Gelmon, Sandra Gelves-Zapata, Gemma Genato, Susan Gent, Natalie Geoghegan, Sam George, Tina George, Simon Georges, Domonique Georgiou, Peter Gerard, Leigh Gerdes, Louise Germain, Helen Gerrish, Abel Getachew, Louise Gethin, Hisham Ghanayem, Anca Gherman, Alison Ghosh, Justin Ghosh, Sudhamay Ghosh, Sarra Giannopoulou, Malick Gibani, Ben Gibbison, Kerry Gibbons, Alex Gibson, Bethan Gibson, Kimberley Gibson, Kirsty Gibson, Sian Gibson, Cat Gilbert, Jeanette Gilbert, Kayleigh Gilbert, Benjamin Giles, Mandy Gill, Lynne Gill, Paul Gillen, Annelies Gillesen, Katherine Gillespie, Elizabeth Gillham, Andrew Gillian, Deborah Gilliland, Robert Gillott, Danielle Gilmour, Kate Gilmour, Franciscus Ginting, Theodora Giokanini-Royal, Anna Gipson, Joanna Girling, Rhian Gisby, Angelena Gkioni, Aikaterini Gkoritsa, Effrossyni Gkrania-Klotsas, Amy Gladwell, James Glanville, Jessica Glasgow, Susannah Glasgow, Jon Glass, Lynn Glass, Sharon Glaysher, Lisa Gledhill, Ana Glennon, John Glover, Kyle Glover, Jan Glover Bengtsson, Chevanthy Gnanalingam, Julie Goddard, Wendy Goddard, Emily Godden, Jo Godden, Emma Godson, Sukanya Gogoi, Aiky Goh, Rebeca Goiriz, Sriya Gokaraju, Raphael Goldacre, Arthur Goldsmith, Portia Goldsmith, Darren Gomersall, Lucia Gomez, Raquel Gomez-Marcos, Ali Gondal, Celia Gonzalez, Jack Goodall, Bob Goodenough, Laura Goodfellow, James Goodlife, Camelia Goodwin, Elizabeth Goodwin, Jayne Goodwin, Paula Goodyear, Rajiv Gooentilleke, Michelle Goonasekara, Sheila Gooseman, Shameer Gopal, Sally Gordon, Hugh Gorick, Caitlin Gorman, Claire Gorman, Stuart Gormely, Diana Gorog, Michelle Gorst, Thomas Gorsuch, Jayshreebahen Gosai, Rebecca Gosling, Sally Gosling, Georgina Gosney, Vanessa Goss, Dzintars Gotham, Naomi Gott, Elizabeth Goudie, Susan Gould, Lysander Gourbault, Abha Govind, Sharon Gowans, Girish Gowda, Rohit Gowda, Hannah Gower, Thomas Gower, Pankaj Goyal, Sunil Goyal, Sushant Goyal, Clive Graham, Jonathan Graham, Justin Graham, Libby Graham, Sharon Graham, Matthew Graham-Brown, Julia Grahamslaw, Gianluca Grana, Tracyanne Grandison, Louis Grandjean, Alison Grant, Ann Grant, David Grant, Matthew Grant, Pauleen Grant, Rhys Gravell, Jenny Graves, Alasdair Gray, Catherine Gray, Georgina Gray, Jackie Gray, Karen Gray, Nicola Gray, Sebastian Gray, Alan Grayson, Fiona Greaves, Paul Greaves, Alastair Green, Charlotte Green, Christopher A Green, David Green, Frederick Green, Joel Green, Marie Green, Nicola Green, Stacey Green, Diarra Greene, Philippa Greenfield, Alan Greenhalgh, Daniel Greenwood, Sandra Greer, James Gregory, Jane Gregory, Katie Gregory, Tamsin Gregory, Jill Greig, Julia Greig, Rebecca Grenfell, Teena Grenier, Susan Grevatt, Glaxy Grey, Andrew Gribbin, Amy Gribble, Natasha Grieg, Douglas Grieve, Ben Griffin, Denise Griffin, Mel Griffin, Sian Griffith, Andrew Griffiths, Daniel Griffiths, David Griffiths, Donna Griffiths, Isabel Griffiths, Mark Griffiths, Nicola Griffiths, Oliver Griffiths, Sarah Griffiths, Yvonne Griffiths, Sofia Grigoriadou, Steph Grigsby, Evelina Grobovaite, Clarissa Grondin, Rachel Groome, Liliana Grosu, Jenny Grounds, Margaret Grout, Helen Grover, Jayne Groves, Neil Grubb, Julie Grundy, Francesca Guarino, Sharada Gudur, Sharazeq Guettari, Shivang Gulati, Vikas Gulia, Pumali Gunasekera, Malin Gunawardena, Kirun Gunganah, Jessica Gunn, Emma Gunter, Alok Gupta, Atul Gupta, Rajeev Gupta, Richa Gupta, Rishi Gupta, Tarun Gupta, Vineet Gupta, Ankur Gupta-Wright, Victoria Guratsky, Alvyda Gureviciute, Sambasivarao Gurram, Anju Gurung, Bhawana Gurung, Shraddha Gurung, Hazel Guth, Pradip Gyanwali, Ruth Habibi, Berkin Hack, Pamela Hackney, Christian Hacon, Aiman Haddad, Denise Hadfield, Michalis Hadjiandreou, Nikolaos Hadjisavvas, Anna Haestier, Nauman Hafiz, Rana Hafiz-Ur-Rehman, Javed Hafsa, Samantha Hagan, Jack William Hague, Rosemary Hague, Kate Haigh, Christina Haines, Scott Hainey, Morton Hair, Brigid Hairsine, Juraj Hajnik, Anne Haldeos, Writaja Halder, Jennie Hale, Carmel Halevy, Paul Halford, William Halford, Alistair Hall, Anthony Hall, Claire Hall, Elizabeth Hall, Fiona Hall, Helen Hall, Jennifer Hall, Kathryn Hall, Jan Hallas, Kyle Hallas, Charles Hallett, Heather Halls, Maryam Hamdollah-Zadeh, Bilal Hameed, Raph Hamers, Raph L Hamers, Imran Hamid, Mohamad Hamie, Bethany Hamilton, Fergus Hamilton, Leigh Hamilton, Nicola Hamilton, Ruth Hamlin, Eleanor Hamlyn, Beatrice Hammans, Shirley Hammersley, Kate Hammerton, Bev Hammond, Leah Hammond, Fiona Hammonds, Ibrahim Hamoodi, Karen Hampshire, Jude Hampson, Lucy Hampson, Ozan Hanci, Sadiyah Hand, Jasmine Handford, Soran Handrean, Sarah Haney, Sheharyar Hanif, E Hanison, Esther Hanison, Jennifer Hannah, Amy Hannington, Merhej Hannun, Aidan Hanrath, Anita Hanson, Jane Hanson, Kathryn Hanson, Steve Hanson, Mazhar Ul Haq, Ala Haqiqi, Monjurul Haque, Lesley Harden, Zoe Harding, Simon Hardman, Joanna Hardy, Kumar Haresh, Rachel Harford, Beverley Hargadon, Carolyn Hargreaves, James Hargreaves, Alice Harin, Mohammed Haris, Edward Harlock, Paula Harman, Tracy Harman, Mark Harmer, Muhammad A Haroon, Charlie Harper, Heather Harper, Peter Harper, Rosemary Harper, Sarah Harrhy, Sian Harrington, Yasmin Harrington-Davies, Jade Harris, Jess Harris, John Harris, Laura Harris, Marie-Clare Harris, Nichola Harris, Sophie Harris, David Harrison, Laura Harrison, Melanie Harrison, Rowan Harrison, Susie Harrison, Thomas Harrison, Wendy Harrison, Elizabeth Harrod, Ciaran Hart, Dominic Hart, Lisa Hartley, Rosemary Hartley, Ruth Hartley, Tom Hartley, William Hartrey, Phillipa Hartridge, Stuart Hartshorn, Alice Harvey, Angela Harvey, Max Harvey, Catherine Harwood, Helen Harwood, Brigitte Haselden, Kazi Hashem, Mohammed Hashimm, Tadaaki Hashimoto, Imranullah Hashmi, Zena Haslam, Adil Hassan, Ali Hassan, Wagae UI Hassan, Waqar Ul Hassan, Sapna Hassasing, Jane Hassell, Philip Hassell, Alex Hastings, Bethany Hastings, Janice Hastings, Jonathan Hatton, May Havinden-Williams, Stefan Havlik, Daniel B Hawcutt, Kadean Hawes, Liz Hawes, Nicola Hawes, Annie Hawkins, Nancy Hawkins, Dan Hawley, Ed Hawley-Jones, Edward Haworth, Cathy Hay, Amna Hayat, Jamal Hayat, Mohamed-Riyal Hayathu, Anne Hayes, Jonas Hayes, Kate Hayes, Melony Hayes, Fiona Hayes, Patrick Hayle, Chloe Haylett, Antara Hayman, Melanie Hayman, Matthew Haynes, Richard Haynes, Rachel Hayre, Sarah Haysom, James Hayward, Patrick Haywood, Tracy Hazelton, Phoebe Hazenberg, Zhengmai He, Elizabeth Headon, Carrie Heal, Brendan Healy, Amy Hearn, Angela Heath, Rowan Heath, Diane Heaton, Kerry Hebbron, Gemma Hector, Andy Hedges, Katrine Hedges, Cheryl Heeley, Elaine Heeney, Rajdeep Heire, Ulla Hemmila, Scott Hemphill, Deborah Hemsley, Abigail Henderson, Jennifer Henderson, Steven Henderson, Joanne Henry, Karol Henry, Lavinia Henry, Margo Henry, Natalie Henry, David Henshall, Gillian Herdman, Rosaleen Herdman-Grant, Morag Herkes, Emma Heron, William Herrington, Emilia Heselden, Peta Heslop, Simon Hester, Emily Hetherington, Joseph Hetherington, Chamila Hettiarachchi, Pramodh Hettiarachchi, Hayley Hewer, John Hewertson, Anna Hewetson, Sue Hewins, Claire Hewitt, Davina Hewitt, Richard Hewitt, Samuel Hey, Robert Heyderman, Mathis Heydtmann, Joseph Heys, Jonathan Heywood, Meg Hibbert, John Hickey, Naomi Hickey, Peter Hickey, Alex Hicks, Jenny Hicks, Scott Rory Hicks, Daniel Higbee, Lucy Higgins, Andrew Higham, Martin Highcock, Judith Highgate, Mondy Hikmat, Amanda Hill, Helen Hill, Joanne Hill, Lisa Hill, Phoebe Hill, Uta Hill, Annette Hilldrith, Catherine Hillman-Cooper, Zoe Hilton, Sarah Hinch, Andrew Hindle, Alice Hindmarsh, Paul Hine, Kim Hinshaw, Clare Hird, Jemma Hives, Benson Ho, Michaela Hoare, David Hobden, Gill Hobden, Maria Hobrok, Simon Hobson, Simon Hodge, Lesley Hodgen, Holly Hodgkins, Louise Hodgkinson, Sally Hodgkinson, David Hodgson, Helen Hodgson, Luke Hodgson, Sheila Hodgson, Gemma Hodkinson, Kenneth Hodson, Matthew Hogben, Lucy Hogg, Lee Hoggett, Abigail Holborow, Catherine Holbrook, Catherine Holden, Melinda Holden, Thomas Holder, Niels Holdhof, Hannah Holdsworth, Lisa Holland, Nicky Holland, Marie Hollands, Elizabeth Holliday, Nina Holling, Laszlo Hollos, Simon Holloway, Marcus Hollyer, Amy Holman, Ann Holmes, Megan Holmes, Raphael Holmes, Rebecca Holmes, Kelly Holroyd, Lyndsey Holt, Siobhan Holt, Susie Holt, Alexandra Holyome, Marie Home, Renate Homewood, Kate Hong, Clare Hooper, Samantha Hope, Susan Hope, Bridget Hopkins, Peter W Horby, Stephanie Horler, Anil Hormis, Daniel Hornan, Nicola Hornby, Zoey Horne, Rebecca Horner, Latoya Horsford, Megan Horsford, Mark Horsford, Valana Horsham, Alexander Horsley, Ashley Horsley, Elizabeth Horsley, Sarah Horton, Jane Hosea, Toby Hoskins, Muhammad Shamim Hossain, Rashed Hossain, Maxine Hough, Sarah Hough, Catherine Houghton, Kathryn Houghton, Rebecca Houlihan, Kay Housely, Hamish Houston, Roseanna Hovvels, Lee How, Laura Howaniec, Laura Howard, Linda Howard, Lucy Howard, Sarah Howard, Stuart Howard, Richard Howard-Griffin, Serena Howe, Mark Howells, Lyn Howie, Kerry Howlett, Sophie Howlett, Josh Hrycaiczuk, Naing Zaya Htoon, Su Htwe, Ying Hu, Chiang Ooi Huah Huah, Abby Huckle, Shahzya Huda, Alison Hudak, Lisa Hudig, Heather Hudson, Oli Hudson, Alison Hufton, Connor Huggins, Alistair Hughes, Emma Hughes, Gareth Hughes, Heather Hughes, Luke Hughes, Rachel Hughes, Rebecca Hughes, Samantha Hughes, Stephen Hughes, Vikki Hughes, Wesley Hughes, Lukas Huhn, Ching Hui, Ruth Hulbert, Diana Hull, Grace Hull, Robert Hull, Amanda Hulme, Peter Hulme, Wendy Hulse, George Hulston, Ryan Hum, Megan Hume, Charlotte Humphrey, Alasdair Humphries, Joanne Humphries, Fiona Hunt, Jonquil Hunt, Kristen Hunt, Luke Hunt, Sophie Hunt, Alexandra Hunter, Karl Hunter, Neil Hunter, George Huntington, Elizabeth Hurditch, Cian Hurley, Katrina Hurley, Mohammed A Husain, Syeda Y Husaini, Coralie Huson, Afreen Hussain, Ibraar Hussain, Ifza Hussain, Mohammad Hussain, Muhammad Hussain, Reda Hussain, Samia Hussain, Sanniah Hussain, Yasmin Hussain, Mohammed Hussam El-Din, Rebecca Hussey, Anja Hutchinson, Camille Hutchinson, Dorothy Hutchinson, Elizabeth Hutchinson, John Hutchinson, Claire Hutsby, Paula Hutton, Thuong Huyen, Daniella Hydes, Jamie Hyde-Wyatt, Niamh Hynes, Megan Hyslop, Mazen Ibraheim, Abdalla Ibrahim, Ahmed Ibrahim, Asil Ibrahim, Mohamed Ibrahim, Wadah Ibrahim, Adetokunbo Idowu Idowu, Muhammad Idrees, Nauman Idrees, Hina Iftikhar, Mawara Iftikhar, Chukwuemeka Igwe, Mohammad Ijaz, Amaju Ikomi, Clare Iles, Stamatina Iliodromiti, Mary Ilsley, Lorna Ilves, La'ali Imam-Gutierrez, Christopher Imray, Haider Imtiaz, Jack Ingham, Julie Ingham, Rory Ingham, Tejas Ingle, Jennifer Inglis, Anne Ingram, Luke Ingram, Peter Inns, Ken Inweregbu, Andreea Alina Ionescu, Ana Ionita, Ilian Petkov Iordanov, Anil Ipe, Madiha Iqbal, Mohammed Iqbal, Faisal Iqbal Sait, Jane Ireland, Robert Irons, Mohannad Irshad, Muhammad S Irshad, Janice Irvine, Val Irvine, Robert Irving, Mina Ishak, Erica Isherwood, Aminul Islam, Samsul Islam, Abdurrahman Islim, Ali Ismail, Omar Ismail, Caroline Ison, M'hamedi Israa, Sharon Isralls, Monica Ivan, Chineze Ivenso, Ashleigh Ivy, Sophie Iwanikiw, Karen Ixer, Menaka Iyer, Mia Iyer, Calum Jack, Amanda Jackson, Ben Jackson, Beth Jackson, Ella Jackson, Helen Jackson, Lauren Jackson, Melanie Jackson, Nicola Jackson, Shane Jackson, Patricia Jacob, Reni Jacob, Nicola Jacques, Anisa Jafar, Daniel Jafferji, Ali Jaffery, Chandrashekar Jagadish, Vijay Jagannathan, Mandeep Jagpal, Fernandez Roman Jaime, Neemisha Jain, Seema Jain, Susan Jain, Sanjay Jaiswal, Danyal Jajbhay, Thomas Jaki, Bintou Jallow, Yusuf Jaly, Sabine Jamal, Zeba Jamal, Yasmin Jameel, Albie James, Christie James, Kate James, Lee James, Linda James, Mark James, Nicholas James, Olivia James, Rebecca James, Ruth James, Tracy James, Jack Jameson, Aaron Jamison, Phoebe Jane, Azara Janmohamed, Sabrina Jansz, Deepa Japp, Victor Jardim, Catherine Jardine, Emma Jarnell, Ellie Jarvie, Claire Jarvis, Rosina Jarvis, Patrycja Jastrzebska, Hafsa Javed, Mays Jawad, Lona Jawaheer, Anu Jayachandran, D Jayachandran, Angelina Jayakumar, Deepak Jayaram, Ravi Jayaram, Geeshath Jayasekera, Thilina Jayatilleke, Abi Jayebalan, Saman Jeddi, Mohammad S Jeelani, Katie Jeffery, Helen Jeffrey, Rachel Jeffrey, Nathan Jeffreys, Benjamin Jeffs, Debbie Jegede, Taylor Jemima, Ifan Jenkin, Alison Jenkins, Christopher Jenkins, David Jenkins, Elinor Jenkins, Sarah Jenkins, Sian Jenkins, Stephen Jenkins, Jacqui Jennings, Louise Jennings, Virginia Jennings, Ellen Jerome, Douglas Jerry, Ellen Jessup-Dunton, Jorge Antonio Jesus Silva, Champa Jetha, Kishan Jethwa, Roshan Jha, Shaman Jhanji, Khoo Jian, Zhixin Jiao, Laura Jimenez, Ana Jimenez Gil, Jithin Jith, Teishel Joefield, Navraj Johal, Karine Johannessen, Aisyah Johari, Annie John, Anu John, Navin John, Emma Johns, Margaret Johns, Antoinette Johnson, Emma Johnson, Gillian Johnson, Kathryn Johnson, Katie Johnson, Luke Johnson, Mark Johnson, Oliver Johnson, Claire Johnston, Janet Johnston, Susan Johnston, Victoria Johnston, Dawn Johnstone, Ed Johnstone, Janet Johnstone, Manohar Joishy, Adam Jones, Alistair Jones, Annabel Jones, Ben Jones, Bryony Jones, Carys Jones, Ceri Jones, Charlotte Jones, Christine E Jones, Debra Jones, Emily Jones, Gareth Jones, Geraldine Jones, Jac Jones, James Jones, Jonathon Jones, Julie Jones, Kate E Jones, Laura Jones, Laura M Jones, Louise Jones, Mathew Jones, Nicola Jones, Paul Jones, Rhianna Jones, Ruth E Jones, Samantha Jones, Sophie Jones, Stefanie Jones, Steve Jones, Taya Jones, Tim Jones, Tracey Jones, Ramya Jonnalagadda, Rebecca Jordache, Sanal Jose, Anna Joseph, P Aiden Joseph, Rosane Joseph, Sibet Joseph, Dhaara Joshi, Mehul Joshi, Pratichi Joshi, Benz Josiah, Tiffany Joyce, Adriel Ju Wen Kwek, Edward Jude, Parminder Judge, Jessica Juhl, Sirisha Jujjavarapu, Mark Juniper, Edmund Juszczak, Deepthi Jyothish, Kasamu Kabiru Dawa, Mark Kacar, Nikhil Kadam, Rebecca Kahari, Gail Kakoullis, Azad Kala Bhushan, Richard J K Kalayi, Roobala Kaliannan Periyasami, Efthymia Kallistrou, Seika Kalsoom, Elisa Kam, John Kamara, Mohamed Kamara, Ajay Kamath, Prakash Kamath, Ravindra Kamath, Siddharth Arun Kamerkar, Nick Kametas, Musaiwale Kamfose, Leia Kane, Osei Kankam, Thogulava Kannan, Abhinav Kant, Vikas Kapil, Ritoo Kapoor, Sonal Kapoor, Sourjya Kar, Janaka Kara, Rona Kark, Abhilasha Karkey, Nicholas Karunaratne, Natashja Kasianczuk, Vidya Kasipandian, Rizwan Kassam, Janarth Kathirgamachelvam, Victoria Katsande, Kulbinder Kaul, Daljit Kaur, Dervinder Kaur, Jasmin Kaur, Jaspreet Kaur, Zunaira Kausar, Mohammad A A Kawser, Andrea Kay, Sarah Kay, Jossy N Kayappurathu, Callum Kaye, Ahemd Kazeem, Rachel Kearns, Nichola Kearsley, Joanne Keating, John Keating, Liza Keating, Elizabeth Keddie-Gray, Natalie Keenan, Jonathan Kefas, Stephen Kegg, Laura Keith, Uzoamaka Keke, Joanne Kellett, Alison Kelly, David Kelly, Diane Kelly, Dominic Kelly, Emma Kelly, Laura Kelly, Martin Kelly, Michael Kelly, Rosalind Kelly, Sinead Kelly, Stephen Kelly, Mary Kelly-Baxter, Marketa Keltos, Timothy Kemp, Alexandra Kendall-Smith, Sarah Kennard, Ann Kennedy, James Kennedy, Sophie Kennedy-Hay, Julia Kenny, Melanie Kent, Lynne Keogan, Alexander Keough, A Kerr, Andrew Kerr, Caroline Kerrison, Anthony Kerry, Helen Kerslake, Ian Kerslake, Helen Kerss, Jocelyn Keshet-Price, Evelyne Kestelyn, Georgina Keyte, Abdul Khadar, Ali Khalid, Muhammad U Khalid, Syed Khalid, Amir Khalil, Asma Khalil, Sijjad Khalil, Abubakar Khan, Ali Khan, Al-Imran Khan, Arham Khan, Asad Khan, Aurangzeb Khan, Burhan Khan, Fatimah Khan, Kausik Khan, Malik Aamaz Khan, Marria Khan, Mehrunnisha Khan, Mohammad Khan, Nayeem Khan, Omar Khan, Rahila Khan, Shabana Khan, Shahul Khan, Shoaib Khan, Tasaduksultan Khan, Waseem Khan, Usman Feroze Khatana, Jibran Khatri, Jyoti Khatri, Hafiza Khatun, Taslima Khatun, Mena Kheia, Jacyntha Khera, Htet Htet Ei Khin, Najaf Khoja, Kiran Khokhar, Chloe Khurana, Faith Kibutu, Andrew Kidd, Michelle Kidd, Joe Kidney, Shane Kidney, Will Kieffer, James Kilbane, Caroline Kilby, Eileen Killen, Susan Kilroy, Bomee Kim, Jee Whang Kim, Sarah Kimber, Andy King, Barbara King, Jennifer King, Kirsten King, Rachel King, Sarah King, Victoria King, Emily King-Oakley, Laura Kingsmore, Fiona Kinney, Sidra Kiran, Jeremy Kirk, Jodie Kirk, Amy Kirkby, Emily Kirkham, Gemma Kirkman, Ursula Kirwan, Toby Kitching, Laura Kitto, Lauren Kittridge, Sarah Klaczek, Frieder Kleemann, Susan Kmachia, Christopher P Knapp, Lucy Knibbs, Alicia Knight, Fraser Knight, Marian Knight, Sarah Knight, Steven Knight, Tom Knight, Ellen Knights, Jane Knights, Martin Knolle, Carol Knott, Charlotte Knowles, Karen Knowles, Laurence Knowles, Emily Knox, Lucy Knox, Oliver Koch, Ronan Kodituwakku, Gouri Koduri, Aisha Koirata, Eirene Kolakaluri, Magdalena Kolodziej, Eirini Kolokouri, Samantha Kon, Niladri Konar, Mari Kononen, Athanasios Konstantinidis, Hui Fen Koo, Imogen Koopmans, Emmanuela Kopyj, Laura Korcierz, James Korolewicz, George Koshy, Chris Kosmidis, Jalpa Kotecha, Easwari Kothandaraman, Koushan Kouranloo, Rukhsana Kousar'c, Margarita Kousteni, Maja Kovac, Alex Kozak Eskenazia, Kestutis Krasauskas, Raghu Krishnamurthy, Vinodh Krishnamurthy, Manju Krishnan, Hari Krishnan, Suzanne Krizak, Sean Krupej, Agnieszka Kubisz-Pudelko, Soren Kudsk-Iversen, Aurimas Kudzinskas, Chirag Kukadiya, Nainesha Kulkarni, Aditi Kumar, Mayur Kumar, Ramesh Kumar, Ravi Kumar, Rita Kumar, Rupa Kumar, Satish Kumar, Vimal Kumar, Arun Kundu, Heinke Kunst, Amit Kurani, Mohammed Kurdy, Rincy Kurian, Vimal Kurmars, Cameron Kuronen-Stewart, Ranganai Scott Kusangaya, Vlad Kushakovsky, Apexa Kuverji, Amma Kyei-Mensah, Thyra Kyere-Diabour, Moe Kyi, Nyan May Kyi, Laura Kyle, Karali-Tsilimpari Kyriaki, Julius Labao, Louise Lacey, Nikki Lack, Emma Ladlow, Heather Lafferty, Shondipon Laha, Sushil Lahane, Clement Lai, James Lai, Robert Laing, Inez Laing-Faiers, Emily Laity, Nicki Lakeman, David Lalloo, Fiona Lalloo, Alison Lam, Fiona Lamb, Lucy Lamb, Thomas Lamb, Pauline Lambert, Claudia Lameirinhas, Mohammed Kadhim Gulam Lami, Holly Lamont, Michal Lamparski, Djillali Lamrani, Christine Lanaghan, Ivone Lancona-Malcolm, Geraldine Landers, Martin J Landray, Matthew Lane, Nicholas Lane, Alidih Lang, Stephen Lang, Daniel Langer, Margaret Langley, Charles Langoya, Emily Langthorne, Taiya Large, Anna Last, Scott Latham, John Latham-Mollart, Afzal Latheef, Nang Latt, Dawn Lau, Eva Lau, Myra Laurenson, Hou Law, Jessica Law, Penny Law, Richard Law, Emma Lawrence, Neil Lawrence, Ryan Lawrie, Louise Lawson, Michael Lay, Christine Laycock, Reina Layug, Maria Lazo, Vietland Le, Amelia Lea, William Lea, Ian Leadbitter, Thomas Leahy, Richard Lean, Lorna Leandro, Darren Leaning, Sandra Leason, Marie Anne Ledingham, Emma Lee, Hannah Lee, Irish Lee, Judith Lee, Sam Lee, Shi Han Lee, Simon Lee, Sindy Lee, Stephanie Lee, Tracey Lee, Xiang Lee, Diana Lees, Jennifer Lees, Helen Legge, Julian Leggett, Katie Leigh-Ellis, Nicky Leitch, Eleni Lekoudis, Petula Lemessy, Nicholas Lemoine, Katy Leng, Katrina Lennon, Liz Lennon, Kelly Leonard, Wen Leong, Nicky Leopold, Oskar Lepiarczyk, Isla Leslie, Eleni Lester, Emma Levell, Chris Levett, Alice Lewin, Alison Lewis, David Lewis, Dee Lewis, Joanne Lewis, Joseph Lewis, Kathryn Lewis, Keir Lewis, Leon Lewis, Marissa Lewis, Rob Lewis, Robert Lewis, Catherine Lewis-Clarke, Katherine Lewiston, Adam Lewszuk, Penny Lewthwaite, Samantha Ley, Angela Liao, Victoria Licence, David Lieberman, Susan Liebeschuetz, Nicky Lightfoot, Patrick Lillie, Ben Lim, Carys Lim, Ee Thong Lim, Ivy Lim, Terence Lim, Wei Shen Lim, Wilson Lim, James Limb, Usha Limbu, Christian Linares, Dermot Linden, Gabriella Lindergard, Kate Lindley, Charlotte Lindsay, Emily Lindsay, Max Lindsay, Helen Lindsay- Clarke, Mirella Ling, Claire Lingam, Linette Linkson, Mike Linney, Louise Linsell, Conrad Lippold, George Lipscomb, Karen Lipscomb, Laura Lipskis, Ana Lisboa, Evangeline Lister, Jeff Little, Sam Little, Xuedi Liu, Daniel K Llanera, Rhiannon Llewellyn, Martin Llewelyn, Adam Lloyd, Aimee Lloyd, Arwel Lloyd, Oliver Lloyd, Richard Lloyd, Su Lo, David Loader, Lydianne Lock, Sara Lock, Stephen Lock, Angela Locke, Jacqueline Locke, Thomas Locke, Teresa Lockett, Jeorghino Lodge, Martina Lofthouse, Heather Loftus, Meg Logan, Chloe A Logue, Sook Yin Loh, Siddharth Lokanathan, Kaatje Lomme, Emily London, Gabriella Long, Natalie Long, Bev Longhurst, Mark Longshaw, Jennifer Lonnen, Caroline Lonsdale, Laura Looby, Ronda Loosley, Paola Lopez, Paula Lopez, Robert Lord, Claire Lorimer, Francesco Loro, Rachel Lorusso, Robert Loveless, Maxine Lovell, Angeliki Loverdou, Andrew Low, Jen Mae Low, Alastair Lowe, Catherine Lowe, Emily Lowe, Faye Lowe, Michael Lowe, Richard Lowsby, Vicki Lowthorpe, Gamu Lubimbi, Alexandra Lubina Solomon, Georgia Lucas, Jacob Lucas, Alice Lucey, Olivia Lucey, Suzanne Luck, H Luke, Jane Luke, Naomi Lungu, Apurva Lunia, Muriel Lunn, Ji Luo, Cindy Nisha Luximon, Barrie Lyell, Elisavet Lyka, Audrey Lynas, Ceri Lynch, Daniel Lynch, Daniella Lynch, Stephen Lynch, Rea-Grace Maamari, Hannah Mabb, Louies Mabelin, Jessica Macaro, Kateryna Macconaill, Chloe Macdonald, Claire Macfadyen, James Gray Macfarlane, Jill Macfarlane, Laura Macfarlane, Lisa MacInnes, Iain MacIntyre, Jill MacIntyre, Kirsten Mack, Callum Mackay, Euan Mackay, Laura Mackay, Alexander Mackenzie, Matt Mackenzie, Robert MacKenzie Ross, Ami Mackey, Fiona Mackie, Robert Mackie, Carolyn Mackinlay, Claire Mackintosh, Katherine Mackintosh, Mary J MacLeod, Michael Macmahon, Andrew MacNair, Catherine Macphee, Iain Macpherson, Catriona Macrae, Allan MacRaild, Alannah Madden, Mary Madden, Norman Madeja, Pradeep Madhivathanan, Madhavi Madhusudhana, Alpha Madu, Lorraine Madziva, Marion Mafham, Nick Magee, Frederick Magezi, Negar Maghsoodi, Christopher Magier, Marios Magriplis, Natasha Mahabir, Subramanian Mahadevan-Bava, Anjanie Maharajh, Kijan Maharjan, Ajit Mahaveer, Bal Mahay, Kanta Mahay, Hibo Mahdi, Thushika Mahendiran, Siva Mahendran, Sarah Maher, Anistta Maheswaran, Shameera Maheswaran, Tina Maheswaran, Parisa Mahjoob-Afag, Ahmed Mahmood, Farhana Mahmood, Waheed Mahmood, Zahra Mahmood, Hager Mahmoud, Ewan Mahony, Luke Mair, Toluwani Majekdunmi, Kesson Majid, Rupert Major, Jaydip Majumdar, Mohammad K H Majumder, Stephen Makin, Marius Malanca, Hannah Malcolm, Flora Malein, Neeraj Malhan, Ayesha Malik, Gulshan Malik, Mohammed Maljk, Paul Mallett, Petrina Mallinder, Georgia Mallison, Louise Mallon, Edward Malone, Gracie Maloney, Madhu Mamman, Irene Man, Kathy Man, Rossana Mancinelli, Marco Mancuso-Marcello, Tracy Manders, Lauren Manderson, Justin Mandeville, Roope Manhas, Carmen Maniero, Ravi Manikonda, Bobby Mann, Jonathan Manning, Pascoe Mannion, Katherine Mansi, Katarina Manso, Dina Mansour, Isheunesu T Mapfunde, Predeesh Mappa, Hemant Maraj, Clare Marchand, Neil Marcus, Maria Marecka, Gomathi Margabanthu, Jordi Margalef, Lavinia Margarit, Georgios Margaritopoulos, Mike Margarson, Fernandez M Maria del Rocio, Teresa Maria Pfyl, Victor Mariano, Ashleigh Maric, Grace Markham, Maria Marks, P Marks, Elisabeth Marouzet, Arran Marriott, Cheryl Marriott, Nemonie Marriott, Karen Marsden, Paul Marsden, Sarah Marsden, Tracy Marsden, Robyn Marsh, Adam Marshall, Andrew Marshall, Gail Marshall, Henry Marshall, Jaimie Marshall, Jenna Marshall, Nicola Marshall, Riley Marshall, Jennifer Marshall, Emmeline Martin, Hayley Martin, Hope Martin, Jane Martin, Karen Martin, Kate Martin, Laila Martin, Michael Martin, Noelia Martin, Tim Martin, Winston Martin, Sarah Martin, Tim Martindale, Marcus Martineau, Lauren Martinez, Jose Carlos Martinez Garrido, Juan Martin-Lazaro, Vijay Kumar Maruthamuthu, Gemma Maryan, Roman Mary-Genetu, Sam Maryosh, Vidan Masani, Diego Maseda, Sheila Mashate, Yasaman Mashhoudi, Al Mashta, Izhaq Masih, Sanna Masih, Nick Maskell, Perry Maskell, Matthew Masoli, Rebecca Mason, Richard Mason, Ruth Mason, Claire Mason, Mohammad Masood, Mohammad Tariq Masood, Syed Masood, Aaqib Masud, Lear Matapure, Cristina Matei, Ropafadzo Matewe, Manraj Matharu, Stephy Mathen, Alex Mather, Nicole Mather, Jonathan Mathers, Joanna Matheson, Amal Mathew, Anna Mathew, Moncy Mathew, Verghese Mathew, Jesha Mathews, Kate Mathias, Darwin Matila, Wadzanai Matimba-Mupaya, Nashaba Matin, Elina Matisa, Max Matonhodze, Elijah Matovu, Jaysankar Mattappillil, Alison Jane Matthews, Heather Matthews, Helen Matthews, Fiona Maxton, Adam Maxwell, Veronica Maxwell, James May, Joanne May, Philippa May, Irving Mayanagao, Matthew Maycock, Graham Mayers, Shelley Mayor, Ibreaheim Mazen, Andrea Mazzella, Nyambura Mburu, Eleanor McAleese, Paul McAlinden, Audrey McAlpine, Graeme McAlpine, Jonathan McAndrew, Hamish McAuley, Sarah McAuliffe, Claire McBrearty, Erin McBride, Michael McBuigan, James McBurney, Laura McCabe, Amanda McCairn, Jake McCammon, Nicole McCammon, Conor McCann, Alexandra McCarrick, Brendan McCarron, Eoghan McCarthy, Michelle McCarthy, Natalie McCarthy, Sinead McCaughey, Gareth McChlery, Tara McClay, Beverley McClelland, Declan McClintock, Patricia McCormack, Jacqueline McCormick, Wendy McCormick, Paul McCourt, Jame McCrae, Sharon McCready, Gordan McCreath, Helen McCreedy, Iain James McCullagh, Liz McCullagh, Megan McCullagh, Conor McCullough, Katherine McCullough, Nicola McCullough, Sarah McCullough, Fiona McCurrach, Rory McDermott, Katharine McDevitt, Helen McDill, Basil McDonald, Claire McDonald, Debbie McDonald, Rob McDonald, Sam McDonald, Damhnaic McDonald, Rowan McDougall, Irene McEleavy, Julie McEntee, Evanna McEvoy, Ruth McEwen, Margaret McFadden, Denise McFarland, Margaret McFarland, Rachel McFarland, Erin McGarry, Lorcan McGarvey, Clodagh McGettigan, Michael McGettrick, Christopher McGhee, Fiona McGill, Sarah McGinnity, Neil McGlinchey, Phil McGlone, Deborah McGlynn, Claire McGoldrick, Clare McGoldrick, Elizabeth McGough, Brendan McGrath, Amanda McGregor, Annemarie McGregor, Heather McGuinness, Sean McGuire, Tara McHugh, Caroline McInnes, Neil McInnes, Karen McIntyre, Mhairi McIntyre, Lorna McKay, Conor Padraig McKeag, Madeleine McKee, Joseph McKeever, Shirley McKenna, Donogh McKeogh, Caroline McKerr, Anthony Michael McKie, Laura Mckie, Gerard McKnight, Heather McLachlan, Andrew McLaren, Barbara McLaren, Nicola McLarty, Maria McLaughlin, James McLay, Mary McLeish, Tina McLennan, Stewart McLure, Anne Marie McMahon, Genevieve McMahon, Mike McMahon, Stephen McMahon, Terence McManus, Moyra McMaster, Paddy McMaster, Samuel McMeekin, Nicola McMillan, Jason McMinn, Liam McMorrow, Helen McNally, Fiona McNeela, Lynne McNeil, Claire McNeill, Shea McNeill, Una McNelis, Melanie McNulty, Roisin McNulty, Christopher McParland, Mark McPhail, Alison McQueen, Anna McSkeane, Denise McSorland, Gini McTaggart, Jacqueline McTaggart, Joanna Mead, Emma Meadows, Olivia Meakin, Ben Mearns, Claire Mearns, Kim Mears, William Mears, Manjula Meda, Ayren Mediana, Ross Medine, Thomas Medveczky, Sharon Meehan, Emily Meeks, Abbi Megan, Nevan Meghani, Salim Meghjee, Rohan Mehra, James Meiring, Rayane Mejri, Sabina Melander, Adriana-Stefania Melinte, Francesca Mellor, Samantha Mellor, Zoe Mellor, Katrina Mellows, Vladimir Melnic, Alice Melville, Julie Melville, Helen Membrey, Mark Mencias, Cheryl Mendonca, Alexander Mentzer, Dan Menzies, Sue Mepham, Oliver Mercer, Pauline Mercer, Arwa Merchant, Fatema Merchant, Mihaela Mercioniu, Megan Meredith, Marta Merida Morillas, Blair Merrick, Jack Merritt, Simon Merritt, Ekta Merwaha, Simon Message, Gabriel Metcalf-Cuenca, Benjamin Metcalfe, Kneale Metcalfe, Stella Metherell, Alexsandra Metryka, Louise Mew, Simon Meyrick, Nhlanhla Mguni, Atiqa Miah, Jagrul Miah, Nahima Miah, Gabriela Mic, Dariush Micallef, Alice Michael, Angiy Michael, Shery Michael, Natalia Michalak, Loredana Michalca-Mason, Janet Middle, Hayley Middleton, Jennifer Tegan Middleton, Maeve Middleton, Sophie Middleton, Shelley Mieres, Loredana Mihalca-Mason, Theresia Mikolasch, Sarah Milgate, Colin Millar, Jonathan Millar, David Miller, Johnathan Miller, Lucy Miller, Rachel Miller, Naomi Miller-Biot, Alex Miller-Fik, Louise Millett, Hazel Milligan, Iain Milligan, Caitlin Milliken, Katherine Millington, Samuel Millington, Helen Mills, Janet Mills, Helen Millward, Rebecca Miln, Alice Milne, Charlotte Milne, Louise Milne, Joanne Milner, Zayar Min, Samuel Mindel, Chrissie Minnis, Paul Minnis, Jane Minton, Frederico Miranda, Lucy Mires, Taimur Mirza, Anjum Misbahuddin, Aseem Mishra, Biswa Mishra, Eleanor Mishra, Ritu Mishra, Sannidhya Misra, Deena Mistry, Heena Mistry, Dushyant Mital, Sarah Mitchard, Alan Mitchell, Ben Mitchell, Piers Mitchell, Philip Mitchelmore, Andrew Mitra, Atideb Mitra, Sandip Mitra, Clarisse Mizzi, Emma Moakes, Emma Moatt, Gita Modgil, Abdelrahman Mohamed, Arez Mohamed, Osab Mohamed, Waheed Mohammad, Aliabdulla Mohammed, Omer Mohammed, Yaser Nabil Sharkawy Mohammed, Bilal Ahmed Mohamud, Amr Moharram, Jonathan Mok, Christine Moller-Christensen, Mateus Mollet, Malid Molloholli, Aoife Molloy, Linda Molloy, Andrew Molyneux, Tasnim Momoniat, Holly Monaghan, Krista Monaghan, Shiva Mongolu, Tesha Monika, Katelyn Monsell, Mahmoud Montasser, Alan Montgomery, Hugh Montgomery, Prebashan Moodley, Margaret Moody, Nick Moody, Angela Moon, James Moon, Ji-Hye Moon, Maria Moon, May Moonan, Parvez Moondi, Alex Moore, Christopher Moore, Davidjar Moore, Faye Moore, Judith Moore, Laura Moore, Sally Moore, Sonia Moore, Rachel Moores, Ed Morab, Jose Morales, Nuria Moramorell, Louise Moran, Grishma Moray, Jeronimo Moreno-Cuesta, Amy Morgan, Caitlin Morgan, Christine Morgan, Colin Morgan, Lauren Morgan, Leila Morgan, Matthew Morgan, Patrick Morgan, Katie Morgan-Jones, Emily Morgan-Smith, Anna Morley, Thomas Morley, Wendy Morley, Anna Morris, Damian Morris, Fiona Morris, Helen Morris, Juliet Morris, Katie Morris, Laura Morris, Lucy Morris, Mary-Anne Morris, Niall Morris, Paul Morris, Sheila Morris, Susan Morris, Douglas Morrison, Moira Morrison, Scott Morrison, Mary Morrissey, Anna Morrow, Franca Morselli, Gordon Mortem, Chelsea Morton, Gordon Morton, Priti Morzaria, Alison Moss, Charlotte Moss, Sarah Moss, Stuart Moss, Nicki Motherwell, Johanna Mouland, Caroline Moulds, Hilary Moulton, Elizabeth Mousley, Karen Moxham, Borja Moya, Quberkani Moyo, Eunice Mshengu, Sheila Mtuwa, Ali Muazzam, Iqtedar Ahmed Muazzam, Nykki Muchenje, Dalia Mudawi, Girish Muddegowda, Imran Mugal, Ahsan Mughal, Javaid Muglu, Javed Muhammad, Carol Muir, Dipak Mukherjee, Syed Asim Ali Mukhtar, Denise Mukimbiri, Peter Mulgrew, Ben Mulhearn, Arafat Mulla, Dee Mullan, Dileepkumar Mullasseril Kutten, Niall Mullen, Rosemary Mullett, Sandra Mulligan, Lana Mumelj, Andrew Mumford, Mohammed Munavvar, Henry Munby, Anne-Marie Munro, Sheila Munt, McDonald Mupudzi, Arshid Murad, Oluwatosin Hakeem Muraina, Koteshwara Muralidhara, Mhairi Murdoch, Jennifer Murira, Alison Murphy, Carl Murphy, Gail Murphy, Peter Murphy, Sheenagh Murphy, Simon Murphy, Clare Murray, David Murray, Eleanor Murray, Katie Murray, Kenneth Murray, Lisa Murray, Lorna Murray, Tracey Murray, Eoin Murtagh, Mithun Murthy, Catherine Murton, Rosie Murton, Neeka Muru, Rosemary Musanhu, Maimuna Mushabe, Omaisa Mushtaq, Ahmed M M Mustafa, Elhaytham Mustafa, Mustafa Mustafa, Ibrahim Mustapha, Zhain Mustufvi, Callum Mutch, Eric Mutema, Balakumar Muthukrishnan, Sheree Mutton, Natasha Muzengi, Memory Mwadeyi, Bettina Mwale, Esther Mwaura, Raji Myagerimath, Alice Myers, Sam Myers, Khin Swe Myint, Yadee Myint, Libor Myslivecek, Evelyn Nadar, Iftikhar Nadeem, Moosa Nadheem, Asma Naeem, Hassan Naeem, Salman Naeem, Samraiz Nafees, Mohamed Nafei, Thapas Nagarajan, Imrun Nagra, Deepak Nagra, Mina Naguib, Kirushthiga Naguleswaran, K Shonit Nagumantry, Kevin Naicker, Sarveshni Naidoo, Gireesha Naik, Rishi Naik, Samir Naik, Devu Sasikumar Nair, Rajiv Nair, Tanushree Nair, Jay Naisbitt, Kerry Naismith, Sri Nallapareddy, Soum Nallapeta, Arumugan Nallasivan, Uttam Nanda, Aarti Nandani, Ali Raza Naqvi, Asadullah Naqvi, Sara Naqvi, Sophia Nasa, Dominic Nash, Nader Nasheed, Abdul Nasimudeen, Umer Nasir, Tahir Nasser, Anuja Natarajan, Geetha Natarajan, Nalin Natarajan, Nikhila Natarajan, Rajkumar Natarajan, Noel Nathaniel, Mala Nathvani, Priyan Nathwani, George Nava, Neena Navaneetham, Jeya Navaratnam, Helen Navarra, Sadaf Naveed, John Navin, Khuteja Nawaz, Sarfaraz Nawaz, Shasta Nawaz, Bonilla Nayar, Suzanne Naylor, Moez Nayyar, Farrah Naz, Mobeena Naz, Babak Nazari, S Nazir, Sehar Nazir, Dumisani Ncomanzi, Onyine Ndefo, Alan Neal, Elaine Neary, Mostafa Negmeldin, Paula Neill, Hector E Neils, Avideah Nejad, Louise Nel, Marie Nelson, Richard Nelson, Scott Nelson, Erni Nelwan, Rajesh Nemane, Samiksha Nepal, Daniel Nethercott, Kimberley Netherton, Kimberley Nettleton, Alison Newby, Angela Newby, David Newby, Tracy Newcombe, Charlotte Newman, Diana Newman, Julie Newman, Oscar Newman, Tabitha Newman, Thomas Newman, Rachel Newport, Christopher Newson, Maria Newton, Anthony Y K C Ng, Ka Wing Ng, Maxine Ng, Sarah Ng, Wee Jin Ng, Thomas Ngan, Gabriel CE Ngui, Alice Ngumo, Caoimhe Nic Fhogartaigh, Nathalie Nicholas, Philip Nicholas, Donna Nicholls, Lisa Nicholls, Alice Nicholson, Anne Nicholson, Annette Nicholson, Ian Nickson, Eileen Nicol, Elizabeth Nicol, Rebecca Nicol, Pantelis Nicola, Antony Nicoll, Pantzaris Nikolaos, Georgii Nikonovich, Annette Nilsson, Kofi Nimako, Louise Nimako, Camus Nimmo, Preethy Ninan, Mahesh Nirmalan, Muhammad Nisar, Toby Nisbett, Aksinya Nisha James, Sabaahat Nishat, Tomoko Nishiyama, Sara Nix, Jennifer Nixon, Maxine Nixon, Khwaja Nizam Ud Din, Maria Nizami, Lyrics Noba, Harriet Noble, Hsu Noe, Jerry Nolan, Zahid Noor, Zaid Noori, Louis Norman, Rachel Norman, Karen Norris, Lillian Norris, Sally Ann Nortcliffe, Fiona North, Julie North, Thomas North, John Northfield, Samantha Northover, Jurgens Nortje, Donna Norton, Rowen Norton, Holly Notman, Khalid Nourein, Timea Novak, Mohamed Nugdallah, Anne Marie Nugent, Justine Nugent, Kribashnie Nundlall, Kieran Nunn, Michelle Nunn, Jane Nunnick, Yvonne Nupa, Zubeir Nurgat, Godfrey Nyamugunduru, Maggie Nyirenda, Kerry Nyland, Daire O Shea, Chloe O'Hara, Kevin O'Reilly, William O'Rourke, Caroline Oakley, Begho Obale, Clements Oboh, Clare O'Brien, Julie O'Brien, Kirsty O'Brien, Linda O'Brien, Neale O'Brien, Rachel O'Brien, Tracey O'Brien, Emma O'Bryan, Ross Obukofe, Christopher O'Callaghan, Lorcan O'Connell, Tadg OConnor, Chris O'Connor, Grainne O'Connor, Miranda Odam, Sam Oddie, Sharon Oddy, Yejide Odedina, Krishma Odedra, Sven Wilhelm Odelberg, Natasha Odell, Omolola Oderinde, Jessica Odone, Catherine O'Donovan, Stephen O'Farrell, Pamela Offord, Tanwa Ogbara, Catherine Ogilvie, Ciaran O'Gorman, Oluwatomilola Ogunkeye, Udeme Ohia, Shinjali Ohja, Ohiowele Ojo, Mark O'Kane, Tolu Okeke, Eleanor OKell, Alicia Okines, Iheoma Okpala, Ernest Okpo, Maryanne Okubanjo, Raphael Olaiya, Tim Old, Gregory Oleszkiewicz, Annie Oliver, Catherine Oliver, Jesse Oliver, Martyn Oliver, Zoe Oliver, Nurudeen O Olokoto, Folusho Olonipile, Olumide Olufuwa, Olatomiwa Olukoya, Akinlolu Oluwole-Ojo, Laura O'Malley, Maryam Omar, Zohra Omar, Nimca Omer, Connaire O'Neill, Lauran O'Neill, Chon Sum Ong, Chidera Onyeagor, Huah Chiang Ooi, Amin Oomatia, Maria Opena, Richard Oram, Christy Ord, Jonathan Ord, Lola Orekoya, Devaki O'Riordan, Sean O'Riordan, Amy Orme, Hannah Orme, Charlotte Orr, Sarah Orr, Christopher Orton, Anna Osadcow, Rawlings Osagie, Rostam Osanlou, Lynn Osborne, Nigel Osborne, Rebecca Osborne, Wendy Osborne, William Osborne, Charles Osbourne, Jennifer Osei-Bobie, Joseph Osman, Wa'el Osman, Bashir Osman, G Osoata, Marlies Ostermann, Eoin O'Sullivan, Susan O'Sullivan, Noor Otey, Otheroro K. Otite, Marie O'Toole, Rachel Owen, Stephanie Owen, Emma Owens, Yetunde Owoseni, Michael Owston, Ruth Oxlade, Feray Ozdes, Jamie Pack, Sophie Packham, Piotr Paczko, Grace Padden, Anand Padmakumar, Iain Page, Joseph Page, Valerie Page, Jodi Paget, Katherine Pagett, Lee Paisley, Susie Pajak, Angela Pakozdi, Soubhik Pal, Sushi Pal, April Palacios, Vishnu Bharadwaj Palagiri Sai, Vadivu Palaniappan, Priya Palanivelu, Adrian Palfreeman, Deepshikha Palit, Alistair Palmer, Lynne Palmer, Ian Pamphlett, Daniel Pan, Anmol Pandey, Nithya Pandian, Krishnaa Pandya, Tej Pandya, Alice Panes, Yee Wei Pang, Laura Pannell, Kanwar Pannu, Suman Pant, Sathianathan Panthakalam, Charles Thomas Pantin, Norman Pao, Helen Papaconstantinou, Padmasayee Papineni, Kitty Paques, Kerry Paradowski, Vinay Parambil, Supathum Paranamana, Siddhant Parashar, Ian Parberry, Amy Parekh, Dhruv Parekh, Louise Parfitt, Helen Parfrey, Omi Parikh, Gemma Parish, John Park, Angela Parker, Ben Parker, Emma Parker, Jacob Parker, Julie Parker, Laura Parker, Lucy Parker, Sara Parker, Sean Parker, Kirstin Parkin, Anna Parkinson, Valerie Parkinson, Chetan Parmar, Viraj Parmar, Victoria Parris, Helen Claire Parry, Siobhan Parslow-Williams, Maria Parsonage, Penny Parsons, Richard Partridge, Kevin Parvin, Lauren Passby, Samuel Passey, Juan Pastrana, Mital Patal, Sarah Patch, Aamie Patel, Alkesh Patel, Amisha Patel, Dakshesh Patel, Darshna Patel, Hemani Patel, Jaymik Patel, Kamal Patel, Kayur Patel, Kiran Patel, Krish Patel, Manish Patel, Martyn Patel, Mehul Patel, Naleem Patel, Nehalbhai Patel, Prital Patel, Saagar Patel, Soonie Patel, Trishna Patel, Vishal Patel, Sangeeta Pathak, Nazima Pathan, Alexandra Patience, Donna Patience, Abigail Patrick, Georgie Patrick, Jean Patrick, Simon Patten, Ben Pattenden, Charlotte Patterson, Linda Patterson, Molly Patterson, Robert Patterson, Leigh Pauls, Stephane Paulus, Amelia Pavely, Susan Pavord, Brendan Payne, Elizabeth Payne, Ruth Payne, Linda Peacock, Louise Peacock, Sarah Peacock, Henry Peake, Jasmine Pearce, Rupert Pearse, Andrew Pearson, Daniel Pearson, Harriet Pearson, Karen Pearson, Samuel Anthony Pearson, Sandra Pearson, Alice Peasley, Hilary Peddie, Russell Peek, Claire Pegg, Suzannah Peglar, Benjamin H Peirce, Claire Pelham, Abigail Pemberton, Melchizedek Penacerrada, Anthony Pender, Carmel Pendlebury, Jessica Pendlebury, Rachel Penfold, Catherine Penman, Julie Penman, Rachel Penman, Justin Penner, Kristi Penney, James Penny, Justin Pepperell, Adriana Pereira, Rita Pereira, Carlota Pereira Dias Alves, Elena Perez, Jane Perez, Tanaraj Perinpanathan, Lakshmi Periyasamy, Elizabeth Perritt, Alison Perry, Emily Perry, Meghan Perry, Thomas M Perumpral, Guilherme Pessoa-Amorim, Ruth Petch, Lionel Peter, Cecilia Peters, Mark Peters, Steve Peters, Tim Peters, Remy Petersen, Alexandra Peterson, Leon Peto, Iulia Petras, Ilianna Petrou, Boyanka Petrova, Mirela Petrova, Paul Pfeffer, Mysore Phanish, Paul Phelan, Christopher Philbey, Jennifer Philbin, Alex Phillips, Dylan Phillips, Rachael Phillips, Marie Phipps, Virach Phongsathorn, Mandeep Phull, Masroor Mubeen Phulpoto, Myat Tin Trafford PI, Sara Pick, James Pickard, Charlotte Pickering, Gillian Pickering, Thomas Pickett, Joanna Pickles, Benjamin Pickwell-Smith, Natalia Pieniazek, Charlie Piercy, Angelo Pieris, Samia Pilgrim, Paul Anthony Pillai, Zoe Pilsworth, Heather Pinches, Stacey Pinches, Kirsty Pine, Muni Tejha Pinjala, Stefania Pintus, Graeme Piper, Tasneem Pirani, Marcus Pittman, Sally Pitts, Nicolene Plaatjies, Naomi Platt, Robert Pleass, Laura Plummer, Charles Plumptre, Jonathan Pobjoy, Tatiana Pogreban, Stephen Poku, Rachel Pollard, Louisa Pollock, Oluwamayowa Poluyi, Gary John Polwarth, Fiona Pomery, Ponmurugan Ponnusamy, Suresh Ponnusamy, Aravind Ponnuswamy, Inês Ponte Bettencourt dos Reis, Suman Pooboni, Alice Poole, Lynda Poole, Michele Poole, Sharon Poon, Tajinder Poonian, David Porter, Jo Porter, Linda Porter, Ross Porter, Kelly Postlethwaite, Narayana Pothina, Priyadarshan Potla, Dorota Potoczna, Jason Pott, Alison Potter, Jean Potter, Sarah Potter, Tracey Potter, Elspeth Potton, Joanne B Potts, Julie Potts, Kathryn Potts, Beli Poudyal, Una Poultney, Katherine Poulton, Vanessa Poustie, James Powell, Jordan Powell, Deborah Power, Nick Power, Joseph Poxon, Robin Poyner, Vidushi Pradhan, Helena Prady, Aalekh Prasad, Krishna Prasad, Fredy Prasanth Raj, Sangeetha Prasath, Anezka Pratley, Steven Pratt, David Preiss, Claire Prendergast, Lynn Prentice, Peter Prentice, Verity Prescott, Laura Presland, Catharine Prest, Stephen Preston, Martha Pretorius, Natalie Prevatt, Sandra Prew, Ashley Price, Carly Price, Claire Price, David Price, Elizabeth Price, Nathan Price, Vivien Price, Anne Priest, Jimena Prieto, Lorraine Primrose, Clare Prince, Judith Prince, Laura Prince, Shirley Pringle, Veronika Pristopan, Kelly Pritchard, Lucy Pritchard, Simon Pritchard, Verma Priyash, Andrew Procter, Clare Proctor, Rebecca Proudfoot, Benjamin Prudon, David Pryor, Solomon Pudi, Joanne Pugh, Lawrence Pugh, Mark Timothy Pugh, Nichola Pugh, Richard Pugh, Veronika Puisa, Kirandip Punia, Saleel Punnilath Abdulsamad, Laura Purandare, Corrina Purdue, Bally Purewal, Molly Pursell, Gregory Purssord, Rory Purves, Sarah Purvis, Khairunnisa Puspatriani, Kathryn Puxty, Zoe Puyrigaud, Michael Pynn, Tariq Qadeer, Mohammad Qayum, Corrine Quah, Sheena Quaid, Nathaniel Quail, Charlotte Quamina, Alice Quayle, Eleanor Quek, Siobhan Quenby, Xinyi Qui, Vanessa Quick, Julie Quigley, Juan-Carlos Quijano-Campos, Andrew Quinn, Tom Quinn, Quratulain Quratulain, Danya Qureshi, Ehsaan Qureshi, Hasanain Qureshi, Khadija Qureshi, Nawaz Qureshi, Qurratulain Qurratulain, Saad Qutab, Muhammad Sharoz Rabbani, Simon Rabinowicz, Madalina Raceala, Raissa Rachman, Laura Rad, Jane Radford, Liz Radford, Jayachandran Radhakrishnan, Cecillia Rafique, Jethin Rafique, Muhammad Rafique, Ravi Ragatha, Aiswarya Raghunathan, Abigail Raguro, Shankho D Raha, Sana Rahama, Mutia Rahardjani, Karen Rahilly, Faisal Rahim, Abdul H Rahimi, Haseena Rana Rahimi, Muhammad Rahman, Salim Ur Rahman, Prajan Rai, Lenka Raisova, Arjun Raj, Pradeep Rajagopalan, Nithy Rajaiah, Arvind Rajasekaran, Aylur Rajasri, Sagar Rajbhandari, Thurkka Rajeswaran, Jyothi Rajeswary, Jeyanthy Rajkanna, Gayathri Rajmohan, Ruth Rallan, Katherine Ralston, Maximilian Ralston, Matsa Ram, Balaji Ramabhadran, Fathima Ramali, Mohamed Ramali, Athimalaipet Ramanan, Shashikira Ramanna, Maheshi Ramasamy, Irfah Rambe, Dhanishta Ramdin, Jozel Ramirez, Mylah Ramirez, Geshwin Ramnarain, Lidia Ramos, Shanthi Ramraj, Alex Ramshaw, Aleem Rana, Ghulam Farid Rana, Rehman Rana, Abby Rand, James Rand, Harpal Randheva, Poonam Ranga, Manmeet Rangar, Harini Rangarajan, Sameer Ranjan, Hannah Rank, Poormina Ranka, Rajesh Rankhelawon, Anita Rao, Sandhya Rao, Sanjay Rao, Deepak Rao, Althaf Abdul Rasheed, Khalid Rashid, Simbisai Ratcliff, Sam Ratcliffe, Sophy Ratcliffe, Sanjeev Rath, Mohmad Iqbal Rather, Selina Rathore, Aravinden Ratnakumar, Jonathan Ratoff, Deepa Rattehalli, Jason Raw, Rachael Raw, Hywel Rawlins, Gautam Ray, Adam Raymond-White, Dana Raynard, Nicola Rayner, Amy Raynsford, Salman Razvi, Zarine Razvi, Kerry Read, Sarah Read, Ajay Reddy, Anvesh Reddy, Harsha Reddy, Ravi Reddy, Aine Redfern-Walsh, Alex Redome, Joan Redome, Anna Reed, John Reed, Andrew Rees, James Rees, Martyn Rees, Sarah Rees, Stephanie Rees, Tabitha Rees, Fiona Regan, Karen Regan, Susan Regan, Kanchan Rege, Ahmed Rehan, A Rehman, Shoib Rehman, Zainab Rehman, Ada Reid, Andrew Reid, Jennifer Reid, Jeremy Reid, Sharon Reid, Mkyla Reilly, Christina Reith, Alda Remegoso, Dinakaran Rengan, Stephen Renshaw, Remya Renu Vattekkat, Henrik Reschreiter, Mark Revels, Glynis Rewitzky, Charles Reynard, Dominic Reynish, Peter Reynolds, Piero Reynolds, Jonathan Rhodes, Naghma Riaz, Emily Rice, Matthew Rice, Mel Rich, Alison Richards, Liz Richards, Suzanne Richards, Celia Richardson, Julie Richardson, Neil Richardson, Nicky Richardson, Joanne Riches, Katie Riches, Leah Richmond, Ruth Richmond, William Ricketts, Hannah Rickman, Anna Riddell, Mohamed Ridha, Carrie Ridley, Paul Ridley, Gudrun Rieck, Linsey Rigby, Samita Rijal, Hannah Riley, Matthew Riley, Phil Riley, Atika Rimainar, Zwesty V P Rimba, Dominic Rimmer, Robert Rintoul, Andrew Riordan, David Ripley, Naomi Rippon, Chloe Rishton, Michael Riste, David Ritchie, Jane Ritchie, Andy Ritchings, Pilar Rivera Ortega, Vanessa Rivers, Batool Rizvi, Syed AS Rizvi, Syed H M Rizvi, James Robb, Ian Roberts, Jane Roberts, Jean Roberts, Karen Roberts, Mark Roberts, Nicky Roberts, Philip Roberts, Rebecca Roberts, Calum Robertson, James Robertson, Jamie Robertson, Nichola Robertson, Stuart Robertson, Nicole Robin, Caroline Robinson, Emma Robinson, Gisela Robinson, Hannah Robinson, Jemima Robinson, Kate Robinson, Matthew Robinson, Ryan Robinson, Sandra Robinson, Steve Robson, Lisa Roche, Samantha Roche, Natalie Rodden, Alistair Roddick, Jack Roddy, Marion Roderick, Alison Rodger, Faye Rodger, Megan Rodger, Alicia Rodgers, Deirdre Rodgers, Natasha Rodgers, Penny Rodgers, Rocio Rodriguez-Belmonte, Nicholas Roe, Charles Roehr, Gill Rogers, Jason Rogers, Joanne Rogers, John Rogers Rogers, Leigh Rogers, Lindsay Rogers, Michaela Rogers, Paula Rogers, Susan Rogers, Thomas Rogers, Paula Rogers, Sakib Rokadiya, Sakib Rokadiya, Lee Rollins, Jennifer Rollo, Catherine Rolls, Claire Rook, Kevin Rooney, Lynsey Rooney, Lace Paulyn Rosaroso, Alastair Rose, Annie Rose, Steve Rose, Zoe Rose, Josephine Rosier, Jack Ross, Jenny Rossdale, Andrew Ross-Parker, Alex Rothman, Joanne Rothwell, Lindsay Roughley, Kathryn Rowan, Neil Rowan, Stephen Rowan, Anna Rowe, Louise Rowe-Leete, Benjamin Rowlands, Megan Rowley, Aparajita Roy, Subarna Roy, Anna Roynon-Reed, Sam Rozewicz, Anna Rudenko, Senthan Rudrakumar, Banu Rudran, Shannon Ruff, Prita Rughani, Sharon Rundell, Jeremy Rushmer, Darren Rusk, Peter Russell, Richard Russell, Cristina Russo, Marieke Rutgers, Aidan Ryan, Brendan Ryan, Lucy Ryan, Matthew Ryan, Pat Ryan, Phil Ryan, Declan Ryan-Wakeling, M Saad, Javeson Sabale, Suganya Sabaretnam, Noman Sadiq, Emma Sadler, Ashiq Saffy, Beth Sage, Harkiran Sagoo, Sobia Sagrir, Rajnish Saha, Sian Saha, Nikhil Sahdev, Sarvjit Sahedra, Jagdeep Sahota, Nooria Said, Sreekanth Sakthi, Hikari Sakuri, Murthy Saladi, Abdul Salam, Armorel Salberg, Erika Salciute, Gina Saleeb, Mumtaz Saleh, Hizni Salih, Laylan Salih, Sarah Salisbury, SiteEneye Saliu, Rustam Salman, Jenny Salmon, Dario Salutous, Mfon Sam, Sally Sam, Tinashe Samakomva, Razan Saman, Sakeena Samar, Renaldo Samlal, Emily Sammons, David Sammut, Mark Sammut, Sunitha Sampath, Claire Sampson, Julia Sampson, Aashna Samson, Anda Samson, Johnson Samuel, Merna Samuel, Reena Samuel, Thomas D L Samuel, Younan Samuel, Elsward Samuels, Theo Samuels, Joanna Samways, Manjula Samyraju, Ilves Sana, Veronica Sanchez, Amada Sanchez Gonzalez, Alina Sanda-Gomez, Peter Sandercock, Amy Sanderson, Tom Sanderson, Prabowo Sandhi, Kuljinder Sandhu, Loveleen Sandhu, Sam Sandow, Victoria Sandrey, Sarah Sands, Mirriam Sangombe, Mathew Sanju, Lakshmi Sankaran, Filipa Santos, Rojy Santosh, Jayanta Sanyal, Aureo F Sanz-Cepero, Dan Saragih, Dinesh Saralaya, Arun Saraswatula, Joshua Sarella, Avishay Sarfatti, Rebecca Sargent, Beatrix Sari, Diah Sari, Khatija Sarkar, Rahuldeb Sarkar, Sruthi Sarma, Zainab Sarwar, Thea Sass, Sonia Sathe, Sobitha Sathianandan, Abilash Sathyanarayanan, Lavanya S J P Sathyanarayanan, Thozhukat Sathyapalan, Prakash Satodia, Vera Saulite, Andrew Saunders, Rachel Saunders, Samantha Saunders, Anne Saunderson, Heather Savill, Karishma Savlani, Gauri Saxena, Matthew Saxton, Amrinder Sayan, Diane Scaletta, Deborah Scanlon, Jeremy Scanlon, Lyndsay Scarratt, Sean Scattergood, Alvin Schadenberg, Jenna Schafers, Wendy Schneblen, Rebecca Schofield, Samuel Schofield, David Scholes, Karen Scholes, Alex Schoolmeesters, Natasha Schumacher, Nicola Schunke, Martin Schuster Bruce, Karin Schwarz, Antonia Scobie, Tim Scorrer, A. Scott, Alistair Scott, Anne Scott, Catherine Scott, Christine Scott, Emily Scott, Kathyn Scott, Leanne Scott, Martha Scott, Stephen Scott, Timothy Scott, Sarah Scourfield, Wendy Scrase, Angela Scullion, Therese Scullion, Emily Seager, Cathy Seagrave, Rebecca Seaman, Eleanor Sear, Isabella Seaton, Anna Seckington, Joanna Sedano, Gabrielle Seddon, Muhammad Adil Seelarbokus, Christopher Sefton, Matias Segovia, Fatima Seidu, Gillian Sekadde, Faye Selby, Georgina Selby, Claire Sellar, Katharine Sellers, Joseph Selley, Victoria Sellick, Gobika Selvadurai, Brintha Selvarajah, Haresh Selvaskandan, Subothini Sara Selvendran, Gary Semple, Nandini Sen, Seema Sen, Aditya Sengupta, Niladri Sengupta, Susana Senra, HoJan Senya, Niranjan Setty, Abigail Seward, Teswaree Sewdin, Jack Seymour, Hussam Shabbir, Fiona Shackley, Tariq Shafi, Aashni Shah, Ahmar Shah, Anand Shah, Bhavni Shah, Momin Shah, Neil Shah, Pallav Shah, Priyank Shah, Qasim Shah, Sarfaraz Hussain Shah, Snehal Shah, Suraj Shah, Syed Shah, Wajid Shah, Saarma Shahad, Sousan Shahi, Sipan Shahnazari, Muhammad Shahzeb, Aisha Shaibu, Zara Shaida, Amina Yousuf Shaikh, Maliha Shaikh, Rajit Shail, Mariya Shaji, Muhammad Shakeel, Korah Shalan, Nadia Shamim, Kazi Shams, Thomas Shanahan, Hamed Sharaf, Muhammad Sharafat, Asir Sharif, Ajay Sharma, Akhilesh Sharma, Ash Sharma, Bhawna Sharma, Mona Sharma, Ojasvi Sharma, Poonam Sharma, Rajeev Sharma, Sanjeev Sharma, Sarkhara Sharma, Shriv Sharma, Sonal Sharma, Alexander Sharp, Charles Sharp, Gemma Sharp, Paula Sharratt, Phoebe Sharratt, Katherine Sharrocks, Christopher Shaw, Daisy Shaw, David Shaw, Deborah Shaw, Joanne Shaw, Jonathan Shaw, Lisa Shaw, Tomos Gruffydd Shaw, Anna Shawcross, Jill Shawe, Lou Shayler, Sophy Shedwell, Jonathan Sheffield, Zak Shehata, Arshiya Sheik, Asif Sheikh, Noorann Sheikh, Benjamin Shelley, Sarah Shelton, Anil Shenoy, Julie Shenton, Amy Shepherd, Kate Shepherd, Lorna Shepherd, Scott Shepherd, Rhian Sheppeard, Helen Sheridan, Ray Sheridan, Samuel Sherridan, Leanne Sherris, Susanna Sherwin, Shaad Shibly, Chiaki Shioi, Anand Shirgaonkar, Kim Shirley, Adebusola Shonubi, Rob Shortman, Rohan Shotton, Sarah Shotton, Ervin Shpuza, Anil Shrestha, Apurba Shrestha, Nora Shrestha, Suchita Shrestha, Karen Shuker, Jack Shurmer, Gilbert Siame, Loria Siamia, Seshnag Siddavaram, Nasir Siddique, Sohail Siddique, Nyma Sikondari, Claudia Silva Moniz, Malcolm Sim, Theresa Simangan, Vimbai Simbi, Robert Sime, Oliver Simmons, Richard Simms, Merritt Simon, Natalie Simon, Angela Simpson, Anna Simpson, Danny Simpson, Georgina Simpson, Joanne Simpson, Kerry Simpson, Phillip Simpson, Thomas Simpson, Kathryn Simpson, Cindy Sing, Ankita Singh, Claire Singh, Jayaprakash Singh, Jyoti Singh, Lokeshwar Singh, Manjeet Singh, Nadira Singh, Pankaj Singh, Prabhsimran Singh, Salil Singh, Saurabh Singh, Parag Singhal, Bryan Singizi, Manas Sinha, Utkarsh Sinha, Guy Sisson, Sarah Sithiravel, Karthikadevi Sivakumar, Shanmugasundaram Sivakumar, Darsh Sivakumran, Sivanthi Sivanadarajah, Pasupathy-Rajah Sivasothy, Nicole Skehan, Robert Skelly, Orlagh Skelton, Imogen Skene, Denise Skinner, Tabitha Skinner, Victoria Skinner, Agnieszka Skorko, Iwona Skorupinska, Mariola Skorupinska, Amy Slack, Katie Slack, Heather Slade, Mark Slade, Lynda Slater, Nicola Slawson, Andrew Sloan, Brendan Sloan, Derek Sloan, Geraldine Sloane, Benjamin Small, Ellen Small, Samuel Small, Karen Denise Smallshaw, Andy Smallwood, Carien Smit, Aileen Smith, Alex Smith, Amanda Smith, Amy Smith, Andrew Smith, Catherine Smith, Chris Smith, Christopher Smith, Dominic Smith, Eleanor Smith, Harriet Smith, Hazel Smith, Helen Smith, Jacky Smith, Jessica Smith, Kate Smith, Kerry Smith, Lara Smith, Linda Smith, Lisa Smith, Loren Smith, Maria Smith, Mel Smith, Oliver Smith, Rachel Smith, Rebecca Smith, Richard Smith, Sally Smith, Samantha Smith, Stacey Smith, Stephanie Smith, Susan Smith, Imogen Smith, John Smith, Sue Smolen, Sara Smuts, Annette Snell, David Snell, Luke Snell, Beng So, Michelle Soan, Toluleyi Sobande, Alberto Sobrino Diaz, Basit Sohail, Bina Sohail, Herminder Sohal, Roy Soiza, Olajumoke Solademi, Babak Soleimani, Amanda Solesbury, Reanne Solly, Louise Solomon, Subash Somalanka, Chandrashekaraiah Somashekar, Raj Sonia, Shiu-Ching Soo, Pavandeep Soor, Germanda Soothill, Jennifer Soren, Apina Sothinathan, Pragalathan Sothirajah, Najwa Soussi, Donna Southam, David Southern, Iain Southern, Louise Southern, Sara Michaela Southin, Jessica Southwell, Thomas Southworth, Jason Sowter, Claudia Spalding, Enti Spata, Katie Spears, Mark Spears, Michelle Spence, Branwell Spencer, Gisele Spencer, Sue Spencer, Tom Spencer, Helen Spickett, Jennifer Spillane, William Spiller, Kerry Spinks, Michelle Spinks, Nick Spittle, Johanna Sporrer, Janet Spriggs, Oliver Spring, Gemma Squires, Jack Squires, Rebecca Squires, Ram Sreenivasan, K Sri Paranthamen, Ramesh Srinivasan, Asha Srirajamadhuveeti, Vino Srirathan, Sybil Stacpoole, Louise Stadon, Jocasta Staines, Nikki Staines, Katie Stammers, Roxana Stanciu, Grazyna Stanczuk, Robyn Staples, Simon Stapley, Natalie Staplin, Adam Stark, Michelle Starr, Rached Stead, Conor Steele, John Steer, Vergnano Stefania, Paula Stefanowska, Caroline Stemp, Alison Stephens, David Stephensen, Elaine Stephenson, Monique Sterrenburg, Melanie Stevens, Will Stevens, Amy Stevenson, Andrew Stevenson, Lesley Stevenson, Sarah Stevenson, Matthew Steward, Claire Stewart, Colin Stewart, McKenna Stewart, Rachel Stewart, Richard Stewart, Jo Stickley, Gemma Stiller, Sarah Stirrup, Sarah Stock, Alexander Stockdale, Lynne Stockham, Paul Stockton, Emma Stoddard, Chris Stokes, Ben Stone, Roisin Stone, Sarah Stone, Imogen Storey, Kim Storton, Frederick Stourton, Angela Strachan, Catherine Strait, Emma Stratton, Jane Stratton, Sam Straw, Dieter Streit, Emma Stride, Sally Stringer, Sophia Strong-Sheldrake, Siske Struik, Carmel Stuart, Anna Stubbs, Harrison Stubbs, Ann Sturdy, Sharon Sturney, Matt Stuttard, Cristina Suarez, Karuna Subba, Christian Peter Subbe, Manjula Subramanian, Venkatram Subramanian, Chinari Subudhi, Rebecca Suckling, Srivatsan Sudershan, Peter Sugden, Rudresh Sukla, Ali Suliman, Fatimah Suliman, Sugrah Sultan, Samyukta Sundar, Reka Sundhar, Edmond Sung, Nadia Sunni, Jay Suntharalingam, Amitava Sur, Dharmic Suresh, Shilpa Suresh, Michael Surtees, C Susan, Danielle Suter, Helen Sutherland, Rachel Sutherland, Rebecca Sutherland, Dovile Sutinyte, Deborah Sutton, Sam Sutton, Mihaela Sutu, Marie-Louise Svensson, Sima Svirpliene, Andrew Swain, Thomas Swaine, Christopher Swales, Tirion Swart, Stephen Sweetman, Ealish Swift, Paul Swift, Pauline Swift, Rachael Swift, Rachel Swingler, Sophie Swinhoe, Katarzyna Swist-Szulik, Luke Swithenbank, Omair Syed, Catriona Sykes, Daisy Sykes, Eliot Sykes, Luke Sylvester, Dominic Symon, Andrew Syndercombe, Zoe Syrimi, Jen Syson, Gemma Szabo, Tamas Szakmany, Megan Szekely, Matthew Szeto, Maria Tadros, Amr Tageldin, Lucy Tague, Hasan Tahir, Muhammad Tahir, Abigail Takyi, Peter Talbot, Alison Talbot -Smith, James Talbot-Ponsonby, Richard Tallent, Bradley Tallon, Adrian Tan, Bee Theng Tan Tan, Hock Tan, Huey Tan, Keith Tan, Wei Teen Tan, Anand Tana, Christina Tanney, Tabitha Tanqueray, Emma Tanton, Anita Tantri, Hayley Tarft, Priyal Taribagil, Obaid Tarin, Syed Tariq, David Tarpey, Lisa Tarrant, Antonia Tasiou, Elizabeth Tatam, Margaret Louise Tate, Kate Tatham, Vera Tavoukjian, Alexander Taylor, Beverley Taylor, Charlie Taylor, David Taylor, Elisabeth Taylor, Janet Taylor, Jennifer Taylor, Joanne Taylor, Julie Taylor, Karen Taylor, Leanne Taylor, Margaret Taylor, Matthew Taylor, Melanie Taylor, Natalie Taylor, Rachael Taylor, Rachel Taylor, Samantha Taylor, Suzanne Taylor, Tracey Taylor, Vicky Taylor, Michelle Taylor-Siddons, Thomas Taynton, Amelia Te, Jessica Teasdale, Julie Tebbutt, Caroline Tee, Rajni Tejwani, Adam Telfer, Vibha Teli, Jennifer Tempany, Julie Temple, Natalie Temple, Helen Tench, Yi He Teoh, Lynne Terrett, Louise Terry, Dariusz Tetla, Shirish Tewari, Daniel Tewkesbury, Joana Texeira, ChiaLing Tey, Clare Thakker, Manish Thakker, Hilary Thatcher, Andrew Thayanandan, Krishna Thazhatheyil, Eaint Thein, Lambrini Theocharidou, Phyu Thet, Kapeendran Thevarajah, Mayooran Thevendra, Nang Thiri Phoo, Yvette Thirlwall, Muthu Thirumaran, Alice Thomas, Andrew Thomas, Caradog Thomas, Emma Thomas, Enson Thomas, Esther Thomas, Helen Thomas, James Thomas, Karen Thomas, Koshy Thomas, Lucy Thomas, Rachel Thomas, Rebecca Thomas, Rhys Thomas, Ruth Thomas, Sarah Thomas, Sherine Thomas, Tessy Thomas, Vicky Thomas, Rhian Thomas-Turner, Catherine Thompson, Christopher Thompson, Clara Thompson, Fiona Thompson, Katharine Thompson, Laura Thompson, Liz Thompson, Luke Thompson, Michael Thompson, Orla Thompson, Rebecca Thompson, Roger Thompson, Nicola Thomson, Natasha Thorn, Charlotte Thorne, Nicola Thorne, Jim Thornton, Richard Thornton, Sara Thornton, Susan Thornton, Thomas Thornton, Tracey Thornton, Christopher Thorpe, Sarah Thorpe, Paradeep Thozthumparambil, Laura Thrasyvoulou, Hannah Thraves, Vicky Thwaiotes, Guy Thwaites, Simon Tiberi, Serena Tieger, Carey Tierney, Caroline Tierney, Mark Tighe, Sorrell Tilbey, Amanda Tiller, John Timerick, Elizabeth Timlick, Alison Timmis, Hayley Timms, Anne-Marie Timoroksa, Samakomva Tinashe, Heather Tinkler, Marianne Tinkler, Jacqui Tipper, Helen Tivenan, Helen T-Michael, Anne Todd, Jackie Todd, Stacy Todd, Mohamed Tohfa, Melanie Tolson, Ana Luisa Tomas, Natalia Tomasova, Sharon Tomlin, Simon Tomlins, Jo Tomlinson, James Tonkin, Ivan Tonna, Catherine Toohey, Kirsty Topham, Mathew Topping, Ruhaif Tousis, Peter Tovey, Gareth Towersey, Jill Townley, Richard Tozer, Helen Tranter, Christopher Travill, Sarah Traynor, Ascanio Tridente, Sanchia Triggs, Fiona Trim, Alex Trimmings, Tom Trinick, Sven Troedson, Emily Tropman, Amy Trotter, Madeleine Trowsdale Stannard, Nigel Trudgill, Maria Truslove, Shaun Trussell, Tariq Trussell, Kyriaki Tsakiridou, Christine Tsang, Peter Tsang, Tan Tsawayo, Kyriaki Karali Tsilimpari, Georgios Tsinaslanidis, Simon Tso, Sally Tucker, Aisha Tufail, Redmond Tully, Grace Tunesi, Killiam Turbitt, Rezon Turel, Tolga Turgut, Claudia Turley, Alison Turnbull, Aine Turner, Ash Turner, Charlotte Turner, Gail Turner, Kate Turner, Kelly Turner, Lucy Turner, Mark Turner, Patricia Turner, Sally Turner, Samantha Turner, Susan Turner, Victoria Turner, Sharon Turney, Jon Turvey, Conor Tweed, David Tweed, Rebecca Twemlow, Emma Twohey, Bhavya Tyagi, Vedang Tyagi, Abigail Tyer, Jayne Tyler, Jennifer Tyler, Alison Tyzack, Petros Tzavaras, Mohammad Saif Uddin, Ruhama Uddin, Ruzena Uddin, Waqar Ul Hassan, Salamat Ullah, Sana Ullah, Sanda Ullah, Athavan Umaipalan, Judith Umeadi, Akudo Umeh, Wilfred Umeojiako, Ben Ummat, Charlotte Underwood, Jonathan Underwood, Adam Unsworth, Veerpal S U Uppal, Gerry Upson, Masood Ur Rasool, Sebastian Urruela, Hiromi Uru, Miranda Usher, Rebecca Usher, Alex UsherRea, Andrew Ustianowski, Linda C Vaccari, Uddhav Vaghela, Abhay Vaidya, Bernardas Valecka, Jennifer Valentine, Balan Valeria, Pramodh Vallabhaneni, Luke Vamplew, Ekaterini Vamvakiti, Joannis Vamvakopoulos, Maud van de Venne, Alex van der Meer, Nora van der Stelt, Joseph Vance-Daniel, Rama Vancheeswaran, Samuel I Vandeyoon, Padma Vankayalapati, Piyush Vanmali, Chloe Vansomeren, William Van't Hoff, Sejal Vara, Stehen J Vardy, Anu Varghese, Maria Varghese, William Varney, Giulia Varnier, Valeria Vasadi, Olivia Vass, Vimal Vasu, Vasanthi Vasudevan, Manu Vatish, Heloyes Vayalaman, Christopher Vaz, Niki Veale, Sachuda Veerasamy, Bar Velan, Swati Velankar, Luxmi Velauthar, Neyme Veli, Nicola Vella, Anitha Velusamy, Ian Venables, Mavi Venditti, Ramya Venkataramakrishnan, Richard Venn, Robert Venn, Lyn Ventilacion, Joanne Vere, Mark Veres, Stefania Vergnano, Will Verling, Amit Verma, Rachel Vernall, Britney Vernon, Mark Vertue, Jerik Verula, Natalie Vethanayagam, Lucy Veys, Carinna Vickers, Saji Victor, Celine Vidaillac, Jennifer Vidler, Bavithra Vijayakumar, Vinod W Vijayaraghavan Nalini, Brigita Vilcinskaite, Neringa Vilimiene, Lynn Vinall, Sylvia Vinay, Latha Vinayakarao, Rachel Vincent, Rosie Vincent, Pritpal Virdee, Emma Virgilio, Abdullah M Virk, Elisa Visentin, Karunakaran Vithian, Sorice Vittoria, Elena Vlad, Ben Vlies, Alain Vuylsteke, Eleftheria Vyras, Richard Wach, Beverley Wadams, Susan Wadd, Natalia Waddington, Phillip Wade, Kirsten Wadsworth, Syed E I Wafa, Daniel Wagstaff, Lynda Wagstaff, Dalia Wahab, Zaroug Wahbi, Abiodun Waheed Adigun, Sawan Waidyanatha, Rachel Wake, Alice Wakefield, William Wakeford, Fiona Wakinshaw, Andrew Walden, Lorna Walding, Alexandria Waldron, Gemma Walker, Harriet Walker, Ian Walker, Kevin Walker, Kim Walker, Linda Walker, Olivia Walker, Rachel Walker, Rebecca Walker, Susan Walker, Rebecca Wallbutton, Jessica Wallen, Karl Wallendszus, Arabella Waller, Rosemary Waller, Gabiel Wallis, Gabriel Wallis, Louise Wallis, Donna Walsh, Elizabeth Walsh, Livia Walsh, Deborah Walstow, Daniel Walter, Alex Walters, Holt Walters, James Walters, Jocelyn Walters, Eileen Walton, Lucy Walton, Olivia Walton, Sharon Walton, Susan Walton, Mandy Wan, Thin Wan, Junita Wanda, Mary Wands, Rachel Wane, Frank Wang, Nick Wang, Ran Wang, Deborah Warbrick, Samantha Warburton, Deborah Ward, Emma Ward, Joanna Ward, Luke Ward, Nicola Ward, Rachael Ward, Thomas Ward, Tom Ward, Scott A Warden, Steve Wardle, Hassan Wardy, Scott Waring, Jenny Warmington, Ben Warner, Christian Warner, Lewis Warnock, Sarah Warran, Jade Warren, Lisa Warren, Yolanda Warren, Hannah Warren-Miell, Gill Warwick, Hazel J Watchorn, Holly Waterfall, Abby Waters, Donald Waters, Mark Waterstone, Catherine Watkins, Catrin Watkins, Eleanor Watkins, Karen Watkins, Lynn Watkins, Abigail Watson, Adam J R Watson, Ekaterina Watson, Eleanor Watson, J G R Watson, Paul Watson, Rebecca Watson, Robert Watson, Malcolm Watters, Donna Watterson, Daniel Watts, John Watts, Merlin Watts, Victoria Waugh, Emma Wayman, Akhlaq Wazir, Mark Weatherhead, Nick Weatherly, Hayley Webb, Kathryn Webb, Kylie Webb, Stephen Webb, Cheryl Websdale, Deborah Webster, Ian Webster, Tim Webster, Ling Wee, Rebecca Weerakoon, Thanuja Weerasinghe, Janaka Weeratunga, Maria Weetman, Shuying Wei, Immo Weichert, Hugh Welch, James Welch, Leanne Welch, Steven Welch, Samantha Weller, Lucy Wellings, Brian Wells, Susan Wellstead, Berni Welsh, Richard Welsh, Ingeborg Welters, Rachael Welton, Lauren Wentworth, Kate Wesseldine, Magdelena West, Raha West, Ruth West, Sophie West, Luke Western, Ruth Westhead, Heather Weston, Alice Westwood, Bill Wetherill, Sharon Wheaver, Helen Wheeler, Ben Whelan, Matthew Whelband, Amanda Whileman, Alison Whitcher, Andrew White, Benjamin White, Christopher White, Duncan White, James White, Jonathan White, Katie White, Marie White, Nick White, Sarah White, Sonia White, Tracey White, Catherine Whitehead, Anne Whitehouse, Claire Whitehouse, Tony Whitehouse, Julia Whiteley, Sophie Whiteley, Gabriel Whitlingum, David Whitmore, Elizabeth Whittaker, Lindsay Whittam, Ashley Whittington, Helen Whittle, Robert Whittle, Eunice Wiafe, Lou Wiblin, Olivia Wickens, John Widdrington, Jason Wieboldt, Hannah Wieringa, Cornelia Wiesender, Laura Wiffen, Andrew Wight, Christopher Wignall, Danielle Wilcock, Emma Wilcock, Louise Wilcox, Laura Wild, Stephen Wild, Michael Wilde, Peter Wilding, Tracey Wildsmith, Joe Wileman, Joy Wiles, Kate Wiles, Elva Wilhelmsen, Thomas Wiliams, Janet Wilkie, David Wilkin, Hannah Wilkins, Joy Wilkins, Suzanne Wilkins, Iain Wilkinson, Lesley Wilkinson, Nicola Wilkinson, Sophia Wilkinson, Susan Wilkinson, Tim Wilkinson, Sylvia Willetts, Aimee Williams, Alexandra Williams, Alison Williams, Angharad Williams, Ava Williams, Carl Williams, Caroline Vivien Williams, Claire Williams, Dewi Williams, Gail Williams, Gemma Williams, Gina Williams, Hannah Williams, James Williams, Jennie Williams, John Williams, Joseph Williams, Karen Williams, Kathryn Williams, Marie Williams, Matthew Williams, Patricia Williams, Penny Williams, Rupert Williams, Samson Williams, Sarah Williams, Sophie Williams, Tamanna Williams, Annie Williamson, Cath Williamson, Catherine Williamson, Dawn Williamson, James D Williamson, Rachel Williamson, Cath Williamson, Helen Williamson, Elizabeth Willis, Emily Willis, Heather Willis, Herika Willis, Joanna Willis, Louise Wills, Lucy Willsher, Catherine Willshire, Francesca Willson, Alison Wilson, Andrea Wilson, Antoinette Wilson, Billy Wilson, James Wilson, Karen Wilson, Kate Wilson, Lucinda Wilson, Mark Wilson, Toni Wilson, Marlar Win, Tin Tin Win, Wut Yee Win Win, Lucinda Winckworth, Laura Winder, Piers Winder, Helen Winmill, Simon Winn, Carmen Winpenny, Helen Winslow, Helen Winter, Jonathan Winter, Barbara Winter-Goodwin, Stephen Wisdom, Matthew Wise, Martin Wiselka, Rebecca Wiseman, Sophie Wiseman, Steven Wishart, Eric Witele, Nicholas Withers, Janet Wittes, Donna Wixted, Therese Wodehouse, Will Wolf, Nicola Wolff, Kirsten Wolffsohn, Rebecca Wolf-Roberts, Elena Wolodimeroff, Adam Wolstencroft, Alan Wong, Charlotte Wong, Chi-Hung Wong, Edwin Wong, Jessica Sue Yi Wong, Kit Y Wong, Mei Yin Wong, Nick Wong, Sam Wong, Amanda Wood, Caroline Wood, Dianne Wood, Fiona Wood, Hannah Wood, Jennifer Wood, Joe Wood, Lisa Wood, Louise Wood, Stephen Wood, Tracy Wood, Katharine Woodall, Rebecca Woodfield, Christopher Woodford, Elizabeth Woodford, Jill Woodford, Louise Woodhead, Timothy Woodhead, Philip Woodland, Marc Woodman, Jane Woods, Katherine Woods, Sarah Woods, Zoe Woodward, Megan Woolcock, Gemma Wooldridge, Rebecca Woolf, Chris Woollard, Christopher Woollard, Louisa Woollen, Emma Woolley, Jade Woolley, Daniel Woosey, Dan Wootton, Joanne Wootton, Daniel Worley, Stephy Worton, Jonathan Wraight, Maria Wray, Kim Wren, Lynn Wren, Caroline Wrey Brown, Catherine Wright, Demi Wright, Francesca Wright, Imogen Wright, Lianne Wright, Rachel Wright, Stephanie Wright, Tim Wright, Caroline Wroe, Hannah Wroe, Henry Wu, Peishan Wu, Pensee Wu, Jonathan Wubetu, Fitria Wulandari, Retno Wulandari, Craig Wyatt, Frederick Wyn-Griffiths, Inez Wynter, Bindhu Xavier, Arnold Xhikola, Zhongyang Xia, Masseh Yakubi, May Yan, Freda Yang, Yingjia Yang, Michael Yanney, Woei Lin Yap, Nabil Yaqoob, Salima Yasmin, Bryan Yates, David Yates, Edward Yates, Helen Yates, Tom Yates, Mark Yates, Charlotte Yearwood Martin, Khin Yein, Fiona Yelnoorkar, Peter Yew, Laura Ylquimiche, Laura Ylquimiche Melly, Inez Ynter, H Yong, Jemma Yorke, Jasmine Youens, Abdel Younes Ibrahim, Eoin Young, Gail Young, Louise Young, Asfand Yousafzar, Sajeda Youssouf, Ahmed Yousuf, Chrissie Yu, Bernard Yung, Daniel Yusef, Said Yusef, Intekhab Yusuf, Anna-Sophia Zafar, Silvia Zagalo, Su Zaher, Aqsa Zahoor, Kareem Zaki, Nabhan Zakir, Kasia Zalewska, Ane Zamalloa, Mohsin Zaman, Shakir Zaman, Julie Zamikula, Louise Zammit, Marie Zammit-Mangion, Esther Zebracki, Daniel Zehnder, Lisa Zeidan, Xiaobei Zhao, Dongling Zheng, Doreen Zhu, Madiha Zia, Omar Zibdeh, Rabia Zill-E-Huma, Ei Thankt Zin, Eleanor Zinkin, Vivian Zinyemba, Christos Zipitis, Arkadiusz Zmierczak, Azam Zubir, Naz Zuhra, Rasha Zulaikha, Sabrina Zulfikar, Carol Zullo, Ana Zuriaga-Alvaro

## Abstract

**Background:**

Aspirin has been proposed as a treatment for COVID-19 on the basis of its anti-thrombotic properties. We aimed to evaluate the efficacy and safety of aspirin in patients admitted to hospital with COVID-19.

**Methods:**

In this randomised, controlled, open-label, platform trial, several possible treatments were compared with usual care in patients hospitalised with COVID-19. The trial took place at 177 hospitals in the UK, two hospitals in Indonesia, and two hospitals in Nepal. Eligible and consenting adults were randomly allocated in a 1:1 ratio to either usual standard of care plus 150 mg aspirin once per day until discharge or usual standard of care alone using web-based simple (unstratified) randomisation with allocation concealment. The primary outcome was 28 day mortality. All analyses were done by intention to treat. The trial is registered with ISRCTN (50189673) and ClinicalTrials.gov (NCT04381936).

**Findings:**

Between Nov 1, 2020, and March 21, 2021, 14 892 (66%) of 22 560 patients enrolled into the RECOVERY trial were eligible to be randomly allocated to aspirin. 7351 patients were randomly allocated (1:1) to receive aspirin and 7541 patients to receive usual care alone. Overall, 1222 (17%) of 7351 patients allocated to aspirin and 1299 (17%) of 7541 patients allocated to usual care died within 28 days (rate ratio 0·96, 95% CI 0·89–1·04; p=0·35). Consistent results were seen in all prespecified subgroups of patients. Patients allocated to aspirin had a slightly shorter duration of hospitalisation (median 8 days, IQR 5 to >28, *vs* 9 days, IQR 5 to >28) and a higher proportion were discharged from hospital alive within 28 days (75% *vs* 74%; rate ratio 1·06, 95% CI 1·02–1·10; p=0·0062). Among patients not on invasive mechanical ventilation at baseline, there was no significant difference in the proportion meeting the composite endpoint of invasive mechanical ventilation or death (21% *vs* 22%; risk ratio 0·96, 95% CI 0·90–1·03; p=0·23). Aspirin use was associated with a reduction in thrombotic events (4·6% *vs* 5·3%; absolute reduction 0·6%, SE 0·4%) and an increase in major bleeding events (1·6% *vs* 1·0%; absolute increase 0·6%, SE 0·2%).

**Interpretation:**

In patients hospitalised with COVID-19, aspirin was not associated with reductions in 28 day mortality or in the risk of progressing to invasive mechanical ventilation or death, but was associated with a small increase in the rate of being discharged alive within 28 days.

**Funding:**

UK Research and Innovation (Medical Research Council), National Institute of Health Research, and the Wellcome Trust through the COVID-19 Therapeutics Accelerator.

## Introduction

Thrombosis is a key feature of severe COVID-19, with 5–30% of hospitalised patients (depending on illness severity) having a major venous thromboembolic event (mostly pulmonary embolism) and up to 3% of patients having an arterial thromboembolic event, particularly myocardial infarction and ischaemic stroke.^1^, ^2^ The risk of thromboembolic complications is reported to be higher in COVID-19 than in other acute medical illnesses and viral respiratory infections, and is associated with worse prognosis.^3^, ^4^

Anti-platelet therapy might have beneficial effects in severe COVID-19 through several mechanisms, including inhibition of platelet aggregation, reduction of platelet-derived inflammation, and blocking of thrombogenic neutrophil extracellular traps.^5^ Aspirin is an affordable, globally available drug which at low doses irreversibly inhibits the cyclooxygenase-1 enzyme, which is responsible for production of thromboxane A2 and proinflammatory prostaglandins. Aspirin can reduce both arterial and venous thrombotic events and has been shown to prevent in-vitro hyperactivity in platelets from patients with SARS-CoV-2.^6^, ^7^ Existing evidence from randomised trials has shown that 75–150 mg aspirin per day is as effective as higher doses in preventing cardiovascular events.^6^

Seven clinical trials of aspirin in COVID-19 are registered, but none have yet reported on the effect of aspirin therapy in COVID-19. Here we report the results of a large randomised controlled trial of aspirin in patients hospitalised with COVID-19.

**Research in context**

**Evidence before this study**

Patients with COVID-19 are at risk of thromboembolic complications. Anti-thrombotic therapies such as aspirin might be useful to prevent vascular events and improve outcomes. We searched MEDLINE, Embase, *bioRxiv, medRxiv*, and the WHO International Clinical Trials Registry Platform (ICTRP), from Sept 1, 2019, up to Feb 25, 2021, for completed published randomised clinical trials establishing the effect of aspirin in patients with COVID-19. For MEDLINE and Embase, we used the search terms “Coronavirus infections/”, “SARS-CoV-2.mp.”, “Coronavirus/” or “CORONAVIRUS.mp”, “COVID.mp.”, “COVID-19.mp.”, “2019-nCoV.mp.”, “COVID19.mp”, “SARSCoV2.mp”, or “SARS-Cov2.mp” and “aspirin.mp”, “aspirin/”, or “acetylsalicylic acid/”, filtered by randomised controlled trials according to validated filters. For *medRxiv* and *bioRxiv*, we used the search term “aspirin”. We identified no published randomised controlled trials assessing aspirin as a treatment for patients with COVID-19 in any clinical scenario. The WHO ICTRP database listed seven ongoing randomised trials of aspirin, two in outpatients and five in inpatients.

**Added value of this study**

To the best of our knowledge, the Randomised Evaluation of COVID-19 Therapy (RECOVERY) trial is the first randomised controlled trial to report on the effect of aspirin as a treatment for hospitalised patients with COVID-19. We found that in 14 892 adults hospitalised with COVID-19, 150 mg aspirin did not reduce 28 day mortality, and among patients who were not receiving invasive mechanical ventilation at randomisation, did not reduce the probability of progression to the composite outcome of invasive mechanical ventilation or death. Allocation to aspirin was associated with an increase in the rate of being discharged alive within 28 days, but the magnitude of the effect was small (1% absolute difference).

**Implications of all the available evidence**

Our findings do not support the use of aspirin as a treatment for hospitalised patients with COVID-19.

## Methods

### Study design and participants

The Randomised Evaluation of COVID-19 Therapy (RECOVERY) trial is an investigator-initiated, individually randomised, controlled, open-label, platform trial to evaluate the effects of potential treatments in patients hospitalised with COVID-19. Details of the trial design and results for other treatments evaluated (including lopinavir–ritonavir, hydroxychloroquine, dexamethasone, azithromycin, tocilizumab, convalescent plasma, and colchicine) have been published previously.^8^, ^9^, ^10^, ^11^, ^12^, ^13^, ^14^ Aspirin comparison was conducted at 167 hospitals in the UK, two hospitals in Indonesia, and two hospitals in Nepal (appendix pp 5–26), and is supported in the UK by the National Institute for Health Research Clinical Research Network. The trial was coordinated by the Nuffield Department of Population Health at the University of Oxford (Oxford, UK), the trial sponsor. The trial was done in accordance with the principles of the International Conference on Harmonisation Good Clinical Practice guidelines and approved by the UK Medicines and Healthcare products Regulatory Agency and the Cambridge East Research Ethics Committee (reference 20/EE/0101). The protocol, statistical analysis plan, and additional information are available on the study website.

Patients admitted to hospital were eligible for the trial if they had clinically suspected or laboratory-confirmed SARS-CoV-2 infection and no medical history that might, in the opinion of the attending clinician, put the patient at substantial risk if they were to participate in the trial. Children younger than 18 years were not eligible for random assignment to aspirin. Patients with known hypersensitivity to aspirin, a recent history of major bleeding, or currently receiving aspirin or another anti-platelet treatment were excluded. Written informed consent was obtained from all patients, or a legal representative if they were too unwell or unable to provide consent.

### Randomisation and masking

Baseline data were collected using a web-based case-report form that included demographics, amount of respiratory support, major comorbidities, suitability of the study treatment for a particular patient, and treatment availability at the study site (appendix pp 33–34). Eligible and consenting adult patients were assigned in a 1:1 ratio to either usual standard of care or usual standard of care plus aspirin using web-based simple (unstratified) randomisation with allocation concealed until after randomisation (appendix p 30). For some patients, aspirin was unavailable at the hospital at the time of enrolment or was considered by the managing physician to be either definitely indicated or definitely contraindicated. These patients were excluded from the randomised comparison between usual care plus aspirin and usual care alone.

As a platform trial, and in a factorial design, patients could be simultaneously randomly assigned to the following other treatment groups: azithromycin, colchicine, or dimethyl fumarate versus usual care; convalescent plasma or casirivimab and imdevimab versus usual care; and baricitinib versus usual care (appendix p 30). Until Jan 24, 2021, the trial also allowed a subsequent randomisation for patients with progressive COVID-19 (evidence of hypoxia and a hyperinflammatory state) to tocilizumab versus usual care. Participants and local study staff were not masked to the allocated treatment. The trial steering committee, investigators, and all other individuals involved in the trial were masked to aggregated outcome data during the trial.

### Procedures

Patients allocated to aspirin received 150 mg by mouth (or nasogastric tube) or by rectum every day until discharge. The 150 mg dose of aspirin once per day was chosen to ensure sufficient inhibition of platelet cyclooxygenase-1 activity in all participants, including those who were overweight.^15^

A single online follow-up form was completed when participants were discharged, had died, or 28 days after randomisation, whichever occurred earliest (appendix pp 35–41). We recorded information on adherence to allocated study treatment, receipt of other COVID-19 treatments, duration of admission, receipt of respiratory or renal support, and vital status (including cause of death). In addition, in the UK, we obtained routine health-care and registry data, including information on vital status (with date and cause of death), discharge from hospital, receipt of respiratory support, or renal replacement therapy.

### Outcomes

Outcomes were assessed 28 days after randomisation, with further analyses specified at 6 months. The primary outcome was all-cause mortality. Secondary outcomes were time to discharge from hospital, and, among patients not on invasive mechanical ventilation at randomisation, progression to invasive mechanical ventilation (including extracorporeal membrane oxygenation) or death. Prespecified subsidiary clinical outcomes were use of non-invasive respiratory support, time to successful cessation of invasive mechanical ventilation (defined as cessation of invasive mechanical ventilation within, and survival to, 28 days), use of renal dialysis or haemofiltration, cause-specific mortality, major bleeding events (defined as intracranial bleeding or bleeding requiring transfusion, endoscopy, surgery, or vasoactive drugs), thrombotic events (defined as acute pulmonary embolism, deep-vein thrombosis, ischaemic stroke, myocardial infarction, or systemic arterial embolism) and major cardiac arrhythmias. Information on suspected serious adverse reactions was collected in an expedited fashion to comply with regulatory requirements.

### Statistical analysis

We did an intention-to-treat comparison between patients randomly assigned to aspirin and patients randomly assigned to usual care but for whom aspirin was both available and suitable as a treatment. For the primary outcome of 28 day mortality, the observed log rank minus the expected statistic and its variance were used to both test the null hypothesis of equal survival curves (ie, the log-rank test) and to calculate the one-step estimate of the average mortality rate ratio. We constructed Kaplan-Meier survival curves to display cumulative mortality over the 28 day period. We used the same method to analyse time to hospital discharge and successful cessation of invasive mechanical ventilation, with patients who died in hospital censored on day 29. Median time to discharge was derived from Kaplan-Meier estimates. For the prespecified composite secondary outcome of progression to invasive mechanical ventilation or death within 28 days (among those not receiving invasive mechanical ventilation at randomisation), and the subsidiary clinical outcomes of receipt of ventilation and use of haemodialysis or haemofiltration, the precise dates were not available, and so the risk ratio was estimated instead.

Prespecified subgroup analyses (defined by characteristics at randomisation, including age, sex, ethnicity, amount of respiratory support, days since symptom onset, and use of corticosteroids) were done for the primary outcome. We did a sensitivity analysis restricting analysis of the primary outcome to patients with a positive PCR test for SARS-COV-2. In addition, we did post-hoc exploratory analyses of the primary and secondary outcomes by venous thromboprophylaxis treatment at randomisation. Observed effects within subgroup categories were compared using a χ^2^ test for heterogeneity or trend, in accordance with the prespecified analysis plan (appendix p 99).

Estimates of rate and risk ratios are shown with 95% CIs. All p values are two sided and are shown without adjustment for multiple testing. The full database is held by the study team that collected the data from the study sites and did the analyses at the Nuffield Department of Population Health, University of Oxford (Oxford, UK).

As stated in the protocol, appropriate sample sizes could not be estimated when the trial was being planned at the start of the COVID-19 pandemic (appendix p 33). As the trial progressed, the trial steering committee, whose members were unaware of the results of the trial comparisons, established that sufficient patients should be enrolled to provide at least 90% power at a two-sided significance level of 1% to detect a clinically relevant proportional reduction in 28 day mortality of 12·5% between the two groups. Consequently, on March 21, 2021, the steering committee, masked to the results, closed recruitment to the aspirin comparison as sufficient patients had been recruited.

Analyses were done using SAS version 9·4 and R version 4·0·3. The trial is registered with ISRCTN (50189673) and ClinicalTrials.gov (NCT04381936).

### Role of the funding source

The funders of the study had no role in study design, data collection, data analysis, data interpretation, or writing of the report.

## Results

**Figure 1:**
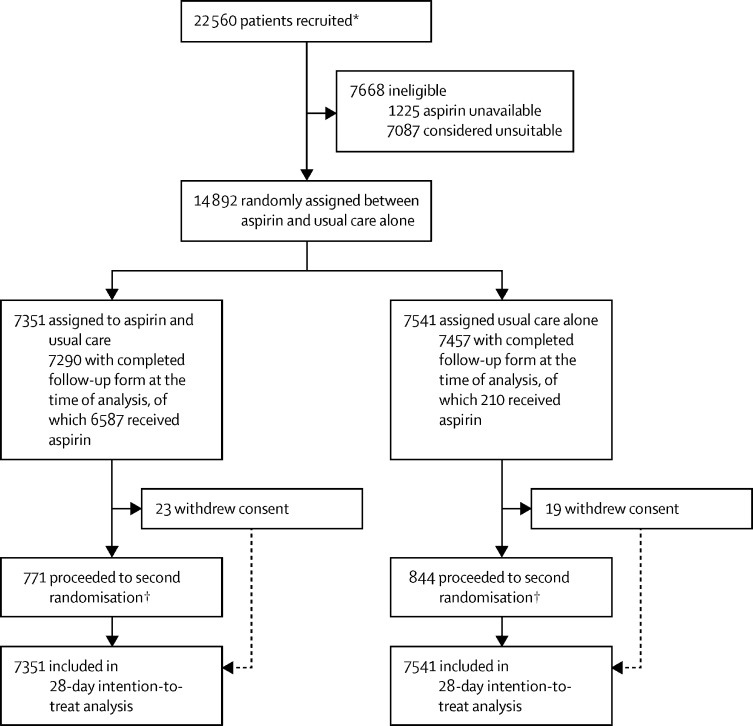
Trial profile Aspirin unavailable and aspirin unsuitable groups are not mutually exclusive. *Number recruited overall during the period that adult participants could be recruited into the aspirin comparison. †Includes 379 (5%) of 7351 patients in the aspirin group and 407 (5%) of 7541 patients in the usual care group allocated to tocilizumab.

Between Nov 1, 2020, and March 21, 2021, 14 892 (66%) of 22 560 patients enrolled into the RECOVERY trial were eligible to be randomly allocated to aspirin (ie, aspirin was available in the hospital at the time and the attending clinician was of the opinion that the patient had no known indication for or contraindication to aspirin; figure 1). Baseline characteristics of the patients are presentend (table 1). 7351 patients were randomly allocated to usual care plus aspirin and 7541 were randomly allocated to usual care alone. The mean age of study participants in this comparison was 59·2 years (SD 14·2) and the median time since symptom onset was 9 days (IQR 6–12 days; appendix p 45). At randomisation, 5035 (34%) patients were receiving thromboprophylaxis with higher-dose low molecular weight heparin (LMWH), 8878 (60%) patients were receiving standard-dose LMWH, and 979 (7%) patients were not receiving thromboprophylaxis (appendix p 54).

**Table 1:** Baseline characteristics

		**Treatment allocation**	
		Aspirin (n=7351)	Usual care (n=7541)
Age (years)		59·2 (14·1)	59·3 (14·3)
	<70	5658 (77%)	5786 (77%)
	70–79	1163 (16%)	1165 (15%)
	≥80	530 (7%)	590 (8%)
Sex			
	Male	4570 (62%)	4631 (61%)
	Female*	2781 (38%)	2910 (39%)
Ethnicity			
	White	5474 (74%)	5655 (75%)
	Black, Asian, and minority ethnic	1176 (16%)	1202 (16%)
	Unknown	701 (10%)	684 (9%)
Number of days since symptom onset		9 (7–12)	9 (6–12)
Number of days since hospitalisation		1 (1–3)	2 (1–3)
Respiratory support received			
	None or simple oxygen	4936 (67%)	5036 (67%)
	Non-invasive ventilation	2057 (28%)	2133 (28%)
	Invasive mechanical ventilation	358 (5%)	372 (5%)
Biochemistry			
	C-reactive protein, mg/L	88 (47–146)	91 (47–150)
	Creatinine, μmol/L	76 (63–93)	76 (62–92)
	D-dimer, ng/mL	475 (205–1088)	489 (210–1083)
Previous diseases			
	Diabetes	1588 (22%)	1659 (22%)
	Heart disease	776 (11%)	788 (10%)
	Chronic lung disease	1425 (19%)	1411 (19%)
	Tuberculosis	20 (<1%)	21 (<1%)
	HIV	25 (<1%)	21 (<1%)
	Severe liver disease†	67 (1%)	53 (1%)
	Severe kidney impairment‡	214 (3%)	251 (3%)
	Any of the previous diseases	3154 (43%)	3247 (43%)
Use of corticosteroids			
	Yes	6906 (94%)	7109 (94%)
	No	441 (6%)	425 (6%)
	Data missing	4 (<1%)	7 (<1%)
SARS-CoV-2 test result			
	Positive	7140 (97%)	7327 (97%)
	Negative	87 (1%)	86 (1%)
	Unknown	124 (2%)	128 (2%)

Data are n (%), mean (SD), or median (IQR).

* Includes 58 pregnant women.

† Defined as requiring ongoing specialist care.

‡ Defined as estimated glomerular filtration rate lower than 30 mL/min per 1·73 m^2^.

The follow-up form was completed for 7290 (99%) of 7351 participants in the aspirin group and 7457 (99%) of 7541 participants in the usual care group. Among participants with a completed follow-up form, 6587 (90%) patients allocated to aspirin received at least one dose of aspirin and 210 (3%) allocated to usual care received at least one dose of aspirin (figure 1; appendix p 46). Of the 6587 participants allocated to aspirin that received at least one dose of aspirin, 5040 (77%) received aspirin on most days following randomisation (≥90% of the days from randomisation to time to discharge or 28 days after randomisation, whichever was earlier). Use of other treatments for COVID-19 was similar among participants allocated to aspirin and among those allocated to usual care, with nearly 90% receiving a corticosteroid, about a quarter receiving remdesivir, and an eighth receiving tocilizumab (appendix p 46).

**Figure 2:**
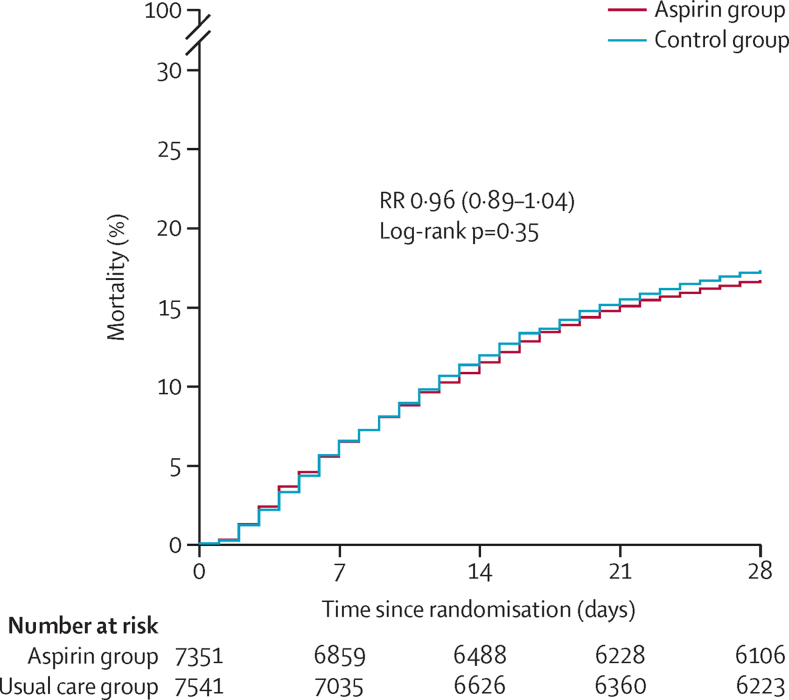
Effect of allocation to aspirin on 28 day mortality RR=rate ratio.

**Figure 3:**
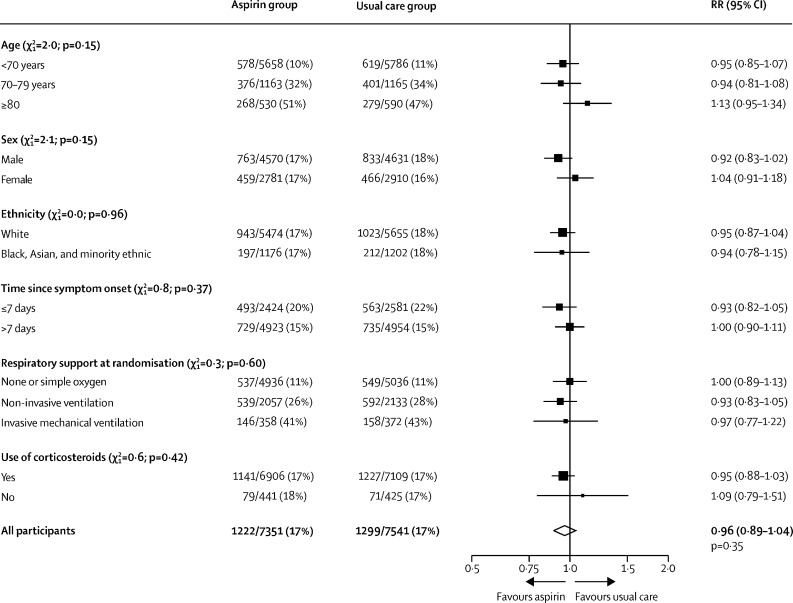
Effect of allocation to aspirin on 28 day mortality by baseline characteristics Subgroup-specific rate ratio estimates are represented by squares (with areas of the squares proportional to the amount of statistical information) and the lines through them correspond to 95% CIs. The ethnicity, days since onset, and use of corticosteroids subgroups exclude patients with missing data, but these patients are included in the overall summary diamond. RR=rate ratio.

Primary and secondary outcome data were known for 99% of randomly assigned patients. We observed no significant difference in the proportion of patients who met the primary outcome of 28 day mortality between the two randomised groups (1222 [17%] of 7351 patients in the aspirin group *vs* 1299 [17%] of 7541 patients in the usual care group; rate ratio 0·96, 95% CI 0·89–1·04; p=0·35; figure 2, table 2). The rate ratio was similar across all prespecified subgroups (figure 3). In an exploratory analysis restricted to the 14 467 (97%) of 14 892 patients with a positive SARS-CoV-2 test result, the result was virtually identical (rate ratio 0·96, 95% CI 0·89–1·04; p=0·31).

**Table 2:** Effect of allocation to aspirin on key study outcomes

	**Treatment allocation**		**RR (95% CI)**	**p value**
	Aspirin (n=7351)	Usual care (n=7541)		
**Primary outcome**				
28 day mortality	1222 (17%)	1299 (17%)	0·96 (0·89–1·04)	0·35
**Secondary outcomes**				
Median time to being discharged alive (IQR), days	8 (5 to >28)	9 (5 to >28)	..	..
Discharged from hospital within 28 days	5496 (75%)	5548 (74%)	1·06 (1·02–1·10)	0·0062
Receipt of invasive mechanical ventilation or death*	1473/6993 (21%)	1569/7169 (22%)	0·96 (0·90–1·03)	0·23
Invasive mechanical ventilation	772/6993 (11%)	829/7169 (12%)	0·95 (0·87–1·05)	0·32
Death	1076/6993 (15%)	1141/7169 (16%)	0·97 (0·90–1·04)	0·39
**Subsidiary clinical outcomes**				
Use of ventilation	1131/4936 (23%)	1198/5036 (24%)	0·96 (0·90–1·03)	0·30
Non-invasive ventilation	1101/4936 (22%)	1162/5036 (23%)	0·97 (0·90–1·04)	0·36
Invasive mechanical ventilation	296/4936 (6%)	325/5036 (6%)	0·93 (0·80–1·08)	0·35
Successful cessation of invasive mechanical ventilation	135/358 (38%)	135/372 (36%)	1·08 (0·85–1·37)	0·54
Renal replacement therapy	273/7291 (4%)	282/7480 (4%)	0·99 (0·84–1·17)	0·93

RR=rate ratio for the outcomes of 28-day mortality and hospital discharge, and rate ratio for the outcome of receipt of invasive mechanical ventilation or death (and its subcomponents).

* Analyses exclude those on invasive mechanical ventilation at randomisation.

Allocation to aspirin was associated with a reduction of 1 day in median time until discharge alive from hospital compared with usual care and an increased rate of discharge alive within 28 days (table 2). Among patients not on invasive mechanical ventilation at baseline, the number of patients progressing to the prespecified composite secondary outcome of invasive mechanical ventilation or death among those allocated to aspirin was similar to that among those allocated to usual care (table 2). There was no evidence that the effect of allocation to aspirin versus usual care on time until discharge alive from hospital, or invasive mechanical ventilation or death differed between the prespecified subgroups of patients (appendix pp 52–53). In a post-hoc exploratory analysis, there was no evidence that the effect of allocation to aspirin versus usual care on the primary and secondary outcomes differed by use of LMWH at randomisation (appendix p 54).

We found no significant differences in the prespecified subsidiary clinical outcomes of cause-specific mortality (appendix p 47), use of ventilation, successful cessation of invasive mechanical ventilation, or receipt of renal dialysis or haemofiltration (table 2). As expected with the use of aspirin, the incidence of thrombotic events was lower (4·6% *vs* 5·3%, absolute difference −0·6%, SE 0·4%) and the incidence of major bleeding events was higher (1·6% *vs* 1·0%, absolute difference 0·6%, SE 0·2%) in the aspirin group (appendix p 48) than in the usual care group. The incidence of new cardiac arrhythmias was similar in the two groups (appendix p 49). There were 18 reports of a serious adverse event believed to be related to aspirin, all of which were due to haemorrhagic events (appendix p 50).

## Discussion

In this large, randomised trial involving more than 14 000 patients and more than 2000 deaths, allocation to aspirin was not associated with reductions in mortality or, among patients not on invasive mechanical ventilation at baseline, the risk of progressing to the composite endpoint of invasive mechanical ventilation or death. Allocation to aspirin was, however, associated with a small increase in the rate of being discharged from hospital alive within 28 days. These results were consistent across the prespecified subgroups of age, sex, ethnicity, duration of symptoms before randomisation, amount of respiratory support at randomisation, and use of corticosteroids.

As expected, allocation to aspirin was associated with an increased risk of major bleeding and a decreased risk of thromboembolic complications, such that for every 1000 patients treated with aspirin, approximately six more patients would have a major bleeding event and approximately six fewer patients would have a thromboembolic event. The rate of reported thromboembolic events in our study population was low (5·3% in the usual care group) in comparison with previous reports.^1^, ^2^ This finding could be because participants were not systematically screened for thromboembolic events, could be related to the widespread use of corticosteroids in the trial population, resulting in reduced thromboinflammatory stimulus, or could be because of the exclusion of patients already receiving aspirin due to previous cardiovascular disease. It is possible that aspirin might have a more meaningful benefit in populations with a higher thrombotic risk, although there would also probably be a corresponding increase in bleeding risk.^16^

The pathogenesis of thromboembolism in COVID-19 is likely to be multifactorial. Coagulopathy is common in severe COVID-19 and is associated with an inflammatory state, neutrophil extracellular traps, and poor outcomes.^2^, ^17^, ^18^, ^19^, ^20^, ^21^ Platelet activation is increased as a result (and potentially by direct interaction with the virus), amplifying inflammation locally and triggering immunothrombosis.^22^, ^23^ In addition, SARS-CoV-2 infection can cause inflammation, dysfunction, and disruption of the vascular endothelium in multiple organs, potentially via direct entry through the angiotensin-converting enzyme 2 receptor.^24^, ^25^, ^26^ The resulting endothelial injury and tissue factor exposure promote thrombosis in the pulmonary circulation and other vascular beds, with microangiopathy and alveolar capillary occlusion contributing to the diffuse alveolar damage and hypoxaemia seen in COVID-19.^25^, ^27^ Furthermore, in autopsy studies pulmonary microthrombi are nine times more frequent in patients with COVID-19 than patients with influenza.^25^

A large number of randomised controlled trials of antithrombotic therapy in COVID-19 are registered, including trials of therapeutic doses of heparin, direct-acting oral anticoagulants, anti-platelet agents, serine protease inhibitors, and thrombolytics.^28^ In patients who are critically ill, the INSPIRATION, REMAP-CAP, ACTIV-4a, and ATTAC trials did not report a benefit in clinical outcomes from therapeutic anticoagulation.^29^, ^30^ Similarly, preliminary results from the COALIZAO-ACTION trial^31^ did not show a benefit from therapeutic anticoagulation (either heparin or rivaroxaban) in a combined endpoint of mortality, successful discharge, or need for oxygen in hospitalised patients with elevated D-dimers. However, the REMAP-CAP, ACTIV-4a, and ATTAC investigators have reported that in patients not critically ill with COVID-19, compared with thromboprophylaxis doses heparin at therapeutic doses was associated with an absolute increase of 4·6% (95% credible interval 0·7–8·1) in the proportion of participants surviving to hospital discharge without receipt of organ support during the first 21 days.^32^

Although there are no other published randomised trial data on the use of aspirin in COVID-19, the REMAP-CAP, ACTIV-4a, and ATTAC report does suggest that antithrombotic therapy might be important in some patients.^32^ The absence of meaningful benefit from aspirin in our trial could be because anti-platelet therapy confers no clinically significant additional benefit on top of high rates of anti-thrombotic therapy with LMWH and corticosteroid treatment diminishing thromboinflammatory stimulation. Alternatively, other non-platelet pathways leading to thrombosis and alveolar damage might be more important determinants of clinical outcomes.

Any potential benefit of antithrombotic therapies in patients with COVID-19 could also depends on timing of treatment initiation, especially if thrombi have already developed by the time of admission.^33^ Thromboembolic events and microthrombi are common in patients with COVID-19 on either prophylactic or therapeutic anticoagulation.^34^ The apparent absence of benefit in INSPIRATION and the REMAP-CAP, ACTIV-4a, and ATTAC severe disease cohorts suggests that these patients might have passed the point at which any benefit from therapeutic anticoagulation could be gained.^29^, ^30^ Although we found no evidence of heterogeneity on the basis of duration of symptoms, baseline disease severity, or background thrombotic prophylaxis regimen, ongoing trials of aspirin in ambulatory populations and those exploring more potent anti-platelet inhibition and fibrinolysis should provide further insights.

The strengths of this trial included that it was randomised, had a large sample size, broad eligibility criteria, and 99% of patients were followed up for the primary outcome. The trial also had some limitations. Detailed information on radiological or physiological outcomes was not collected. Although this randomised trial is open label (ie, participants and local hospital staff are aware of the assigned treatment), the primary and secondary outcomes are unambiguous and were ascertained without bias through linkage to routine health records. However, it cannot be excluded that reporting of thromboembolic and bleeding events might have been influenced by knowledge of treatment allocation. Nevertheless, the proportional effects of aspirin on these events were very similar to those reported in previous large clinical trials of aspirin in people with previous cardiovascular disease.^6^

The RECOVERY trial only studied patients with COVID-19 who were hospitalised, and therefore is not able to provide evidence on the safety and efficacy of aspirin used in other patient groups. Further studies to identify the safety and efficacy of aspirin in patients with COVID-19 who are not hospitalised are needed and are ongoing.

In summary, the results of this large, randomised trial do not support the addition of aspirin to standard thromboprophylaxis or therapeutic anticoagulation in patients hospitalised with COVID-19.

## Data sharing

The protocol, consent form, statistical analysis plan, definition and derivation of clinical characteristics and outcomes, training materials, regulatory documents, and other relevant study materials are available online at https://www.recoverytrial.net. As described in the protocol, the trial Steering Committee will facilitate the use of the study data and approval will not be unreasonably withheld. Deidentified participant data will be made available to researchers registered with an appropriate institution within 3 months of publication. However, the Steering Committee will need to be satisfied that any proposed publication is of high quality, honours the commitments made to the study participants in the consent documentation and ethical approvals, and is compliant with relevant legal and regulatory requirements (eg, relating to data protection and privacy). The Steering Committee will have the right to review and comment on any draft manuscripts before publication. Data will be made available in line with the policy and procedures described at https://www.ndph.ox.ac.uk/data-access. Individuals wishing to request access should complete the form at https://www.ndph.ox.ac.uk/files/about/data_access_enquiry_form_13_6_2019.docx and e-mail data.access@ndph.ox.ac.uk.

## Declaration of interests

The authors declare no competing interests or financial relationships relevant to the submitted work. No form of payment was given to anyone to produce the manuscript. The Nuffield Department of Population Health at the University of Oxford has a staff policy of not accepting honoraria or consultancy fees directly or indirectly from industry.
